# NetMIM: network-based multi-omics integration with block missingness for biomarker selection and disease outcome prediction

**DOI:** 10.1093/bib/bbae454

**Published:** 2024-09-17

**Authors:** Bencong Zhu, Zhen Zhang, Suet Yi Leung, Xiaodan Fan

**Affiliations:** Department of Statistics, The Chinese University of Hong Kong, Shatin, New Territories, Hong Kong SAR, China; Department of Statistics, The Chinese University of Hong Kong, Shatin, New Territories, Hong Kong SAR, China; Department of Pathology, School of Clinical Medicine, LKS Faculty of Medicine, The University of Hong Kong, Queen Mary Hospital, Hong Kong SAR, China; Department of Statistics, The Chinese University of Hong Kong, Shatin, New Territories, Hong Kong SAR, China

**Keywords:** multi-omics integrative, Markov random field, block missingness, data augmentation

## Abstract

Compared with analyzing omics data from a single platform, an integrative analysis of multi-omics data provides a more comprehensive understanding of the regulatory relationships among biological features associated with complex diseases. However, most existing frameworks for integrative analysis overlook two crucial aspects of multi-omics data. Firstly, they neglect the known dependencies among biological features that exist in highly credible biological databases. Secondly, most existing integrative frameworks just simply remove the subjects without full omics data to handle block missingness, resulting in decreasing statistical power. To overcome these issues, we propose a network-based integrative Bayesian framework for biomarker selection and disease outcome prediction based on multi-omics data. Our framework utilizes Dirac spike-and-slab variable selection prior to identifying a small subset of biomarkers. The incorporation of gene pathway information improves the interpretability of feature selection. Furthermore, with the strategy in the FBM (stand for ”full Bayesian model with missingness”) model where missing omics data are augmented via a mechanistic model, our framework handles block missingness in multi-omics data via a data augmentation approach. The real application illustrates that our approach, which incorporates existing gene pathway information and includes subjects without DNA methylation data, results in more interpretable feature selection results and more accurate predictions.

## Introduction

Recent rapid developments of high-throughput technologies have made it feasible to explore an individual patient’s genome through long lists of genetic, epigenetic, and transcript features. Consequently, integrative analysis based on such multi-level omics data, also known as vertical integration, has gained significant attention as a means of understanding the fundamental mechanism of disease and pathologies [1, 2]. Compared with research focused on a single type of genomic alteration, an integrative framework simultaneously considers the regulatory mechanism between different omics data, thereby enhancing our comprehension of the causality of disease and the interaction between environments and patients [3, 4].

Multi-omics data integration methods have been used widely for several crucial tasks in bioinformatics. For the clustering task, several methods have been developed, including iCluster [5] and its variants [6, 7], Bayesian consensus clustering [8], and BayesianTWL [9], to cluster cancer patients based on multi-omics data. For association studies, frequentist approaches like collaborative regression [10], canonical variate regression [11], LRM-SVD [12], DFNForest [13], and deep learning methods [14] are utilized for outcome prediction, classification, and feature selection through penalty-based techniques. In the Bayesian framework, iBAG [15], FBM [16], and a Bayesian negative binomial mixture regression model [17] are employed to investigate the regulatory pattern between gene expression and DNA methylation, as well as the association between gene expression and clinical outcomes.

One major challenge in multi-omics integration is the high dimensionality of measured features. Most of the above-mentioned methods tackle the high-dimensionality problem through dimension reduction techniques [18] or feature selection techniques. Feature selection is enabled by popular methods like penalization methods such as LASSO [19], elastic net [20], and fused LASSO [21], as well as Bayesian variable selection priors like Bayesian LASSO [22] and spike-and-slab priors [23, 24]. To evaluate the performance of different methods, some benchmark studies have also been developed [25–27].

Although feature selection methods have been successful, few of the aforementioned techniques consider the known structural dependencies among genetic features. Consequently, the results obtained are hard to interpret in downstream enrichment analysis, as genes in the same pathway often have similar cellular functions. To address these limitations and obtain more consistent results, several methods have been developed that incorporate known network or pathway information. For example, Gao *et al*. (2019) [28] identified significant genetic features via a network-constrained regularization, while Stingo and Vannucci (2011) [29] incorporated pathway information through the Markov random field (MRF) prior to the variable selection of discriminant analysis. Li *et al*. (2022) [30] also incorporated prior network information through penalization in the clustering of messenger RNA (mRNA) expression data. However, these frameworks mainly focus on single omics data, and a unified framework that integrates known network and pathway information for multi-omics data is still limited.

**Figure 1:**
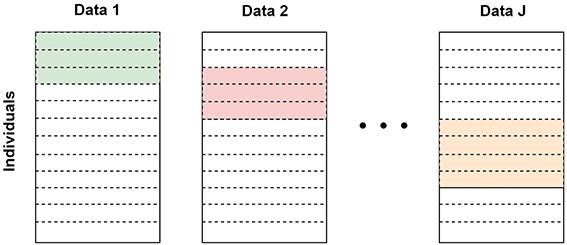
Major missing pattern in multi-omics data; each row consists of blocks representing different omics data types from the same individual; the colored blocks highlight the areas where some omics data are missing for certain individuals.

Another challenge in multi-omics integrative analysis is the high proportion of missing data, particularly in the block missing structure (Fig.1), where only a portion of the subjects are measured in all types of platforms [16]. For example, in The Cancer Genome Atlas (TCGA) kidney cancer study’s kidney renal clear cell carcinoma (KIRC) project, 532 cancer subjects were measured in gene expression, but only 319 cancer subjects were measured in DNA methylation with the 450k array. Most integrative approaches only consider the complete data case by removing subjects without all omics data types. However, this naive approach decreases the sample size, resulting in reduced statistical power, particularly when the number of types of omics data is high. To address the block missing problem, integrative imputation techniques are conducted before model fitting, which utilize the correlations and shared information among multi-omics data sets, such as canonical correlation analysis [31] and multiple imputations of multiple factor analysis [32]. In contrast, a Bayesian framework can offer a unified framework for handling missing data. In the Bayesian analysis dealing with missing data, one approach involves modeling the marginal distribution of observed data after integrating out the missing observations when the missing mechanism is missing at random [33]. The other approach, data augmentation, samples missing data from their conditional posterior distribution [34, 35]. For example, FBM [16] employs data augmentation to accomplish the block missing problem. However, it shows low efficiency in feature selection and lacks interpretability due to the absence of dependency structure information among the features. To the best of our knowledge, there is no unified framework that handles missing data while also serving the network-based feature selection and model prediction in multi-omics data integration.

Motivated by the challenges in multi-omics data analysis and the iBAG model, which constructs the relationship between gene expression and DNA methylation *via* a mechanistic model, we proposed the Network-based Multi-omics Integration with Missingness (NetMIM) method to address the interpretability problem and situations where only partial subjects have all types of omics data. Compared with another iBAG-driven model (FBM), which only focuses on continuous response and provides unsatisfactory feature selection results, our proposed framework makes several contributions. Firstly, it utilizes Dirac spike-and-slab variable selection prior to identifying a small subset of biomarkers associated with clinical outcomes. Secondly, it incorporates the known structural dependencies among biomarkers into feature selection via an MRF prior, improving the interpretability of feature selection. In the simulation analysis, we show that the informative prior can improve the performance of feature selection and model prediction. Lastly, the novel unified framework is extended to handle binary response and survival response while effectively addressing missing data through a data augmentation approach.

The article is structured as follows. In Section 3, we introduce our Bayesian integrative analysis model and the inference of parameters. Section 3 comprises extensive simulations and comparisons with other methods in various scenarios. In Section 3, we evaluate the proposed method on multi-omics data with continuous response. In Section 3, we present real applications to KIRC and lung adenocarcinoma (LUAD) datasets from TCGA, where a proportion of subjects have no DNA methylation. Finally, in Section 3, we provide our conclusions and discussions.

## Methods

Let $\boldsymbol{Y}_{N}$ denote the clinic outcome of interest for a total of $N$ patients, the type of which could be a continuous response, dichotomous response, and survival response. For the $n$-th patient, $(m_{n1}, m_{n2}, \dots , m_{nJ})$ represents the measurement of $J$ DNA methylation probes and $(e_{n1}, e_{n2}, \ldots , e_{nK})$ represents the mRNA expression levels for $K$ genes. Apart from genomic features, $L$ clinic covariates, including age, gender, and tumor stage, are denoted as $ (c_{n1}, c_{n2}, \ldots , c_{nL})$. Hence, all the data can be denoted as $\{\boldsymbol{Y}_{N},\mathbf{M}_{N\times J}, \mathbf{E}_{N \times K}, \mathbf{C}_{N \times L} \}$ in matrix notation.

### Model

The structure of NetMIM is motivated by the iBAG model [15] which integrates multi-omics data according to the biological mechanism. It is known that molecular features measured at the transcript level (e.g. mRNA expression) affect clinical outcomes more directly than molecular features measured at the DNA/epigenetics level (e.g. DNA methylation). Molecular features measured at the DNA level affect clinical outcomes by influencing mRNA expression. Under the guidance of these biological mechanisms, the iBAG model partitions gene expression into different (independent) units and uses this to identify genes relevant to clinical outcomes as modulated by other DNA level features, including DNA methylation.

**Figure 2:**
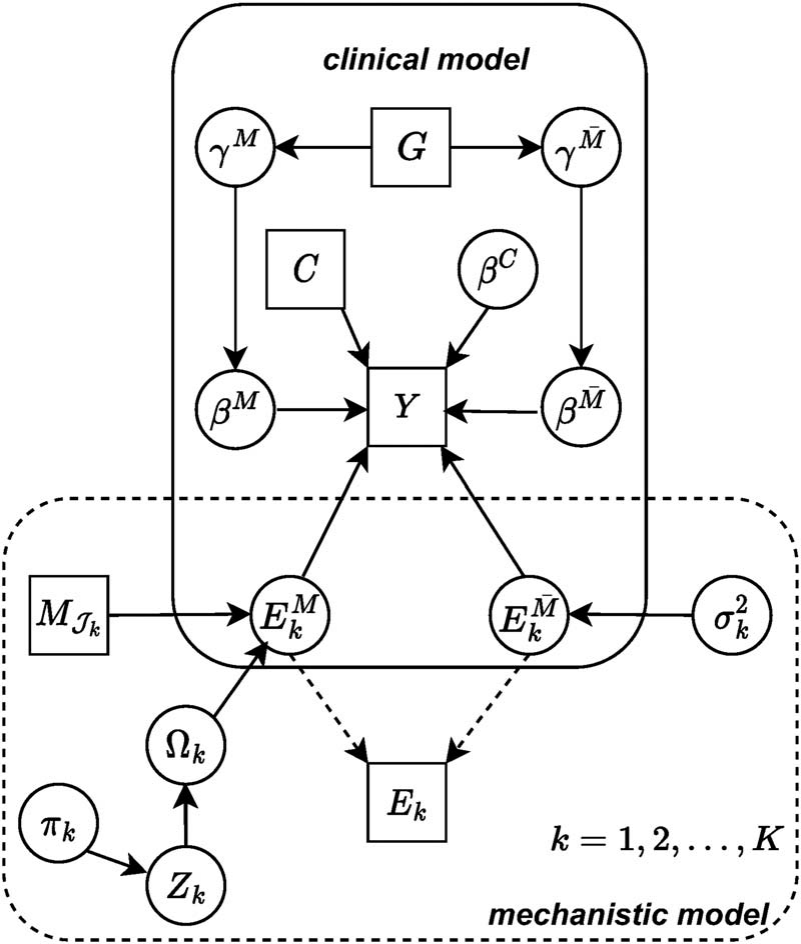
A graphical representation of the proposed model; each node in a circle is a parameter of the model and each node in a square represents an observable information; the solid line refers to a probabilistic direct dependence and the dashed line refers to a deterministic relationship; the hyperparameters are omitted.

NetMIM contains a two-layer hierarchical structure (Fig. 2). The first layer is a *mechanistic* model to infer the effect of DNA methylation on gene expressions. In the mechanistic model, the gene expressions are decomposed into the part modulated by methylation ($\mathbf{E}^{M}$) and the part controlled by other regulators ($\mathbf{E}^{\bar{M}}$). 


(1) \begin{align*}& \mathbf{E} = \mathbf{E}^{M} + \mathbf{E}^{\bar{M}}, \quad \mathbf{E}^{M} = \mathbf{M}\Omega,\end{align*}

where $\mathbf{E}^{M} = (e^{M}_{nk})_{N \times K} = (\boldsymbol{E}^{M}_{1}, \dots , \boldsymbol{E}^{M}_{K})$: $\boldsymbol{E}^{M}_{k}$ denotes the effect of DNA methylation on $k$-th gene expression; $\mathbf{E}^{\bar{M}} = (e^{\bar{M}}_{nk})_{N \times K} = (\boldsymbol{E}^{\bar{M}}_{1}, \dots , \boldsymbol{E}^{\bar{M}}_{K})$:$\boldsymbol{E}^{\bar{M}}_{k}$ denotes the effect of other regulators on $k$-th gene expression; $\Omega = (\omega _{jk})_{J \times K}$: $\omega _{jk}$ is the ”gene-methylation” effect, indicating the effect of $j$-th methylation probe on the $k$-th gene.

The second layer is a *clinical* model to assess the relationship between clinical outcome and genomic information obtained in the mechanistic model. For continuous response, the model is 


(2) \begin{align*}& \boldsymbol{Y} = \mathbf{C}\boldsymbol{\beta}^{C} + \mathbf{E}^{M}\boldsymbol{\beta}^{M} + \mathbf{E}^{\bar{M}} \boldsymbol{\beta}^{\bar{M}} + \boldsymbol{\epsilon},\end{align*}

where $\boldsymbol{\beta }^{C} = (\beta ^{C}_{1}, \dots , \beta ^{C}_{L})$ denotes the effects of clinical factors on the outcome; $\boldsymbol{\beta }^{M} = (\beta ^{M}_{1}, \ldots , \beta _{K}^{M})$ denotes the effects of the part of the gene expressions regulated by DNA methylation, called *type M effect*; $\boldsymbol{\beta }^{\bar{M}} = (\beta ^{\bar{M}}_{1}, \ldots , \beta _{K}^{\bar{M}})$ denotes the effects of the part of the gene expressions regulated by other mechanisms, called *type $\bar{M}$ effect*. The error term $\boldsymbol{\epsilon }$ is assumed to follow the Gaussian distribution, given by $N(\boldsymbol{0}, \sigma ^{2} \mathbf{I}_{N})$. A normal prior $N(0, \tau _{c}^{-1}\sigma ^{2})$ is assigned on $\beta _{l}^{C}$ for $l \in \{1, \ldots , L\}$.

In the original iBAG model, all methylation sites/probes within the promoter region of a given gene are summarized to generate the gene’s single methylation level. However, since not all methylation probes regulate gene expression, it is more appropriate to include methylation probes in the promoter region with a shrinkage prior on the regression coefficients. Therefore, we specify a *spike-and-slab* prior on the ”gene-methylation” effect $\Omega $: 


(3) \begin{align*}& \omega_{jk} | z_{jk}, \sigma_{k}^{2} \sim (1-z_{jk})\mathcal{\delta}_{0}(\omega_{jk}) + z_{jk} \mathcal{N}(0, \tau_{k}^{-1}\sigma_{k}^{2}),\end{align*}

where $z_{jk}$ is the binary indicator for $j$-th methylation probe, $\sigma ^{2}_{k}$ is the variance of mechanistic model for $k$-th gene, and $\mathcal{\delta }_{0}(\cdot )$ is the Dirac delta function. A simple independent Bernoulli prior, $z_{jk} \sim \text{Bern}(\pi _{k})$, is assigned, where $\pi _{k}$ could be either treated as a hyperparameter or a random variable. We assign a Beta distribution $\text{Beta}(a, b)$ on $\pi _{k}$, leading to a Beta-Binomial prior on the number of effective methylation probes.

**Figure 4:**
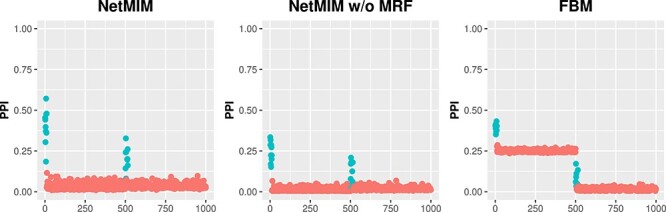
Posterior inclusion probabilities of features for the three Bayesian methods when $K = 500$ and sd $=2$; the x-axis is the feature index, among which the first $500$ indices are $\mathbf{E}^{\bar{M}}$ variables and the second $500$ indices are $\mathbf{E}^{M}$ variables; the blue points represent the effective features and the red points represent the ineffective features; the PPI values for features are the average based on 50 replicated simulated data.

Due to the high dimensionality of genes, it is necessary to consider variable selection approaches in the clinical model. We specify a similar Dirac spike-and-slab prior [17] on *type M effect* and *type $\bar{M}$ effect* to induce sparsity: 


(4) \begin{align*}& \begin{aligned} \beta^{M}_{k} | \gamma_{k}^{M}, \sigma^{2} \sim (1-\gamma_{k}^{M}) \mathcal{\delta}_{0}(\beta^{M}_{k}) + \gamma_{k}^{M} \mathcal{N}(0, \tau^{-1}\sigma^{2}), \\ \beta^{\bar{M}}_{k} | \gamma_{k}^{\bar{M}}, \sigma^{2} \sim (1-\gamma_{k}^{\bar{M}}) \mathcal{\delta}_{0}(\beta^{\bar{M}}_{k}) + \gamma_{k}^{\bar{M}} \mathcal{N}(0, \tau^{-1}\sigma^{2}), \end{aligned}\end{align*}

where $\gamma _{k}^{M}$ and $\gamma _{k}^{\bar{M}}$ are binary indicators, indicating whether or not there exists *type M effect* or *type $\bar{M}$ effect* for $k$-th gene. Compared with another iBAG-driven method FBM [16], which utilized a continuous spike-and-slab distribution for same level variable selection in Equation (3) and (4), our model can achieve zero coefficient exactly with Dirac distribution. More interestingly, the continuous spike-and-slab prior tends to falsely include $\mathbf{E}^{\bar{M}}$ variables, preventing to identify the effect of $\mathbf{E}^{M}$ (see Fig. 4).

Although it is feasible to assign an independent Bernoulli prior on the indicators, recent contributions in Bayesian variable selection of modeling genomic features have incorporated the external information about dependencies among variables via MRF priors [17, 29]. Hence, we specify an MRF prior on $\boldsymbol{\gamma }$, considering dependencies among genes that are represented by a gene–gene interaction network extracted from the KEGG database. It is given by 


(5) \begin{align*}& p(\gamma_{k}|\boldsymbol{\gamma}_{-k}) = \frac{\exp\left(\gamma_{k}(d + f \sum_{k^{^{\prime}} \in N_{k}} \gamma_{k^{\prime}} )\right)}{1 + \exp\left(d + f \sum_{k^{\prime} \in N_{k}} \gamma_{k^{\prime}} \right)},\end{align*}

where $d$ and $f$ are hyperparameters, $\boldsymbol{\gamma }_{-k}$ denotes the vector of $\boldsymbol{\gamma }$ with $k$-th element excluded, and $N_{k}$ is the set of neighbors of $k$-th element in the network. $d$ is negative, encouraging sparsity in the model, and $f$ is positive, indicating neighboring elements are jointly effective in the model. For the node without any neighbor, its prior distribution reduces to a Bernoulli prior with parameter $\pi = \exp (d)/(1+\exp (d))$. The joint prior on $\gamma $ is 


(6) \begin{align*}& p(\boldsymbol{\gamma}) \propto \exp \left(d \mathbf{1}_{1 \times K} \boldsymbol{\gamma}+f \boldsymbol{\gamma}^{T} \boldsymbol{G} \boldsymbol{\gamma}\right),\end{align*}

where $\boldsymbol{G}$ is $p \times p$ binary symmetric matrix representing the links in the gene network. If gene $j$ and $l$ are linked in the network, we have $g_{jl} = 1$; otherwise $g_{jl} = 0$. Since the expression of gene $k$ is decomposed into two parts, $\boldsymbol{E}^{M}_{k}$ and $\boldsymbol{E}^{\bar{M}}_{k}$, we set $\gamma _{k} = \gamma _{k}^{M} \vee \gamma _{k}^{\bar{M}}$, which means that any one of components being effective implies the gene $k$ is effective in the model. We complete the model by assigning an inverse-Gamma prior on the variance parameters in the model given by $\sigma ^{-2} \sim \text{Ga}(\delta _{1}, \delta _{2})$ and $\sigma ^{-2}_{k} \sim \text{Ga}(\delta _{1}, \delta _{2})$.

### Model with missingness

In Bayesian frameworks, when data are missing at random, one approach is to model the marginal distribution of observed data by integrating out missing data. The other approach is the data augmentation method, which imputes the missing values from the conditional distribution of observed data and current parameters in each iteration. Following the strategy of handling missing data in the FBM model [16], the NetMIM model utilizes the data augmentation method to handle missing omics data types.

Let $\boldsymbol{U}^{E} = (U_{1}^{E}, \ldots , U_{N}^{E})$ be the missing indicator of gene expression data and $\boldsymbol{U}^{M} = (U_{1}^{M}, \ldots , U_{N}^{M})$ be the missing indicator of DNA methylation; we assume $U_{i}^{E} \times U_{i}^{M} \neq 1 $ for $ \forall 1 \leq i \leq N$, indicating the patient should have at least one type of omics data. The full omics data contain the observed part and missing part, denoted, respectively, by $\mathbf{E}_{N \times K} = (\mathbf{E}^{t}_{N_{obs}^{E} \times K}, \mathbf{E}^{t}_{N_{mis}^{E} \times K})^{t}$ and $\mathbf{M}_{N \times J} = (\mathbf{M}^{t}_{N_{obs}^{M} \times J}, \mathbf{M}^{t}_{N_{mis}^{M} \times J})^{t}$, where $N^{E}_{obs} = N - \sum _{i=1}^{N} U_{i}^{E}$ and $N_{obs}^{M} = N - \sum _{i=1}^{N} U_{i}^{M}$. For the expression of $k$-th gene in the missing data, we specify the following model: 


(7) \begin{align*}& \boldsymbol{E}_{N^{E}_{mis}, k} \sim N(\mathbf{M}_{N^{E}_{mis},\mathcal{J}_{k}} \omega_{k}, \sigma_{k}^{2} \mathbf{I}_{N^{E}_{mis} \times N^{E}_{mis}}),\end{align*}

where $\mathcal{J}_{k}$ is the set of methylation probes mapped to the $k$-th gene.

Since we only consider the methylation probes in the promoter region of each gene, the methylation probes are many-to-one mapped to genes, meaning that many methylation probes are mapped to one gene, but one methylation probe is only mapped to one gene. For the DNA methylation of the $j$-th probe in the missing data, we impose the imputation model by 


(8) \begin{align*}& \boldsymbol{M}_{N_{mis}^{M}, j} \sim N\left(0, (\sigma^{m}_{j})^{2}\mathbf{I}_{N^{M}_{mis} \times N^{M}_{mis}}\right),\end{align*}

where $(\sigma ^{m}_{j})^{2}$ is the variance for the $j$-th methylation probe. $(\sigma ^{m}_{j})^{2} = 1$ if the M value of DNA methylation data is normalized with mean $0$ and variance $1$.

### Model for discrete and survival outcomes

Following the latent variable formulation in the iBAG [15], the NetMIM model can be easily extended to model discrete and censored outcomes. Specifically, when the clinical response is a binary variable taking values of 0 or 1, we can augment a latent continuous variable $Y^{*}$ via the probit model. The relationship between the latent continuous variable $Y_{n}^{*}$ and $Y_{n}$ can be expressed as 


(9) \begin{align*}& Y_{n}= \begin{cases} 1 & \text{ if}\ Y_{n}^{*}>0 \\ 0 & \text{ otherwise} \end{cases}\end{align*}

for $n = 1, \ldots , N$. The response in Equation (2) is replaced by $Y^{*}$ and parameter representations and corresponding interpretations remain the same as those for continuous outcomes.

If the clinical outcome of interest is patient survival time (with censoring), we use the accelerated failure time model [36]. For right-censored response variable $Y_{n} = (y_{n}, \delta _{n})$, where $y_{n} = \min (t_{n}, c_{n})$ is the minimum value between survival time $t_{n}$ and censoring time $c_{n}$ of patient $n$, and $\delta _{n} = \mathcal{I}\{t_{n} \leq c_{n}\}$ is event indicator. The relationship between augmented continuous variable $Y^{*}$ and response variable $Y$ can be expressed as 


(10) \begin{align*}& \begin{cases}\log \left(y_{n}\right)=Y_{n}^{*} & \text{ if } \delta_{n}=1 \\ \log \left(y_{n}\right) < Y_{n}^{*} & \text{ if } \delta_{n}=0\end{cases}.\end{align*}

The full conditionals and the Markov chain Monte Carlo (MCMC) sampling schemes for discrete and survival responses are provided in the Supplementary Material.

### Model fitting and posterior inference

We detailed an MCMC algorithm based on the stochastic search for variable selection and Gibbs sampler for parameter estimation in the Supplementary Material. For posterior inference, our primary interest lies in the selection of genes associated with clinical response, as captured by the indicators $\boldsymbol{\gamma }^{M}$ and $\boldsymbol{\gamma }^{\bar{M}}$. To summarize the posterior distribution of model selection indicators, one approach is to use *maximum-a-posterior* estimates. However, due to the large model space, an alternative approach is preferred, which involves thresholding the estimated marginal posterior probability of inclusion (PPI) of a single feature [17]. The PPI is obtained as the proportion of included MCMC iterations after burn-in where the corresponding feature is selected within the model. For the choice of threshold, the procedure to control the Bayesian false discovery rate proposed by Newton *et al*. (2004) [37] could be applied. It is suggested that a threshold equal to $0.5$ often results in a reasonable Bayesian FDR, so we follow their rule by selecting features with PPI larger than $0.5$ [38].

Our second interest is the prediction of clinical response using the Bayesian model average approach. Given a new sample $\boldsymbol{x}_{new} = (\boldsymbol{C}_{new}, \boldsymbol{E}_{new}, \boldsymbol{M}_{new})$ in the validation data set, the prediction of response in the $i$-th iteration is $\hat{y}_{i} = \boldsymbol{C}_{new}\hat{\beta }^{c}_{i} + \boldsymbol{M}_{new} \cdot \hat{\Omega }_{i}\hat{\beta }^{M}_{i} + (\boldsymbol{E}_{new} - \boldsymbol{M}_{new} \cdot \hat{\Omega }_{i}) \hat{\beta }^{\bar{M}}_{i}$, where $\hat{\beta }^{c}_{i}$, $\hat{\beta }^{M}_{i}$, $\hat{\beta }^{\bar{M}}_{i}$, and $\hat{\Omega }_{i}$ are the simulated parameters from the $i$-th MCMC iteration. We average the estimation from $T - T_{b}$ iterations, obtaining $\hat{y}_{new} = (\sum _{i=T_{b}+1}^{T} \hat{y}_{i})/(T-T_{b})$, where $T_{b}$ is the burn in iteration. For continuous response, the predicted mean square error (PMSE) will be calculated by 


(11) \begin{align*}& PMSE = \sqrt{\sum_{i=1}^{N_{v}}(y_{new, i}- \hat{y}_{new, i})^{2}/N_{v}},\end{align*}

where $N_{v}$ is the sample size of the validation data set.

We use the area under the curve (AUC) of the receiver operating characteristic, which is a plot of the true positive rate (TPR) versus the false positive rate (FPR), to evaluate the effectiveness of feature selection. We utilize the PPI values of the features in simulations where the underlying truth is known. By summarizing the relationship between TPR and FPR under different PPI thresholds, the AUC provides a more accurate measurement for feature selection, ranging from $0$ to $1$. A higher AUC indicates a more accurate feature selection. To assess the performance of outcome prediction, we calculate the PMSE on the independent test datasets in simulations and the real application with continuous response. A smaller PMSE indicates a better outcome prediction. In the real application where the clinical response is a right-censored variable associated with the patient’s survival time (KIRC and LUAD), we utilize the concordance index (C-index) [39] as the metric to evaluate outcome prediction. The C-index is defined as the proportion of patient pairs in which the predictions and outcomes are concordant.

## Simulation

### Simulation schemes

To evaluate the performance of our NetMIM model, we conduct several simulations based on full omics data and missing omics data according to the schemes in FBM [16]. Specifically, the clinical covariate matrix is simulated from $N(0,1)$ with $N = 100$ and $L = 3$. The number of genes $K \in \{100, 200, 500\}$. To preserve the realistic pattern of correlation in DNA methylation, we randomly select $J = 300$ methylation probes from DNA methylation of real data. The average number of methylation probes mapped to each gene is $3$. Each methylation probe is randomly allocated to a gene with the constraint that each gene contains at least one methylation probe. In the mechanistic model, $50\%$ of genes are randomly selected to be significantly regulated by their methylation probes, and $z_{jk} \sim \text{Bern}(1/2)$ for $j \in \mathcal{J}_{k}$, the methylation probe set in the promoter region of gene $K$, when the $k$-th gene is significantly regulated by DNA methylation. For the selected methylation probes, the coefficient $w_{jk}$ is sampled from $\text{Unif}(0.5, 1)$ or $\text{Unif}(-1, -0.5)$, and the regulated part $\mathbf{E}^{M} = \mathbf{M}\Omega $. Then the residual part $\mathbf{E}^{\bar{M}}$ is sampled from $N(\boldsymbol 0, \text{diag}(\sigma ^{2}_{1:K}))$ with $\sigma ^{2}_{1} = \cdots =\sigma ^{2}_{K} = 1$. The gene expression matrix $\mathbf{E} =\mathbf{E}^{\bar{M}} + \mathbf{E}^{M}$. In the clinical model, the first five genes are selected to influence the clinical outcome through both the part regulated by methylation and the part not regulated by methylation. The second five genes are selected to impact the outcome only through the part regulated by DNA methylation, and the third five genes impact through the part not regulated by methylation. It implies that both $\boldsymbol{\gamma }^{M}$ and $\boldsymbol{\gamma }^{\bar{M}}$ have $10$ out of $K$ genes equal to one, and the remaining are zeros. We simulate the graph structure by sampling edges among effective genes from $\text{Bern}(0.1)$ and sampling the same number of edges among ineffective genes. For the effective genes, the regression coefficients in the clinical model are sampled from $\text{Unif}(1, 1.5)$ or $\text{Unif}(-1.5, -1)$. The coefficients of clinical covariates and intercept are set as $1$, and the variance of clinical model $\sigma ^{2} \in \{1, 4, 9\}$ is used to generate outcome $Y$. In each simulation, $100$ independent samples are generated as the validation dataset.

In scenario I, we evaluate the proposed method (NetMIM), the proposed method without MRF prior (NetMIM w/o MRF), the FBM method [16], FBMcorr (FBM method considering correlation in DNA methylation), and two mimic frequentist methods, mimic lasso and mimic elastic net, in the simulated full omics data with varying variances and different numbers of genes. In NetMIM without MRF, we set the hyperparameter $f = 0$ in the MRF prior. The mimic lasso and mimic elastic net methods are implemented by modeling each gene expression by the corresponding methylation probes via lasso [19] and elastic net [20] regression, respectively. After the parts of gene expression regulated by methylation are obtained from the previous procedure, the clinical model is also modeled through lasso and elastic net regression. The hyperparameters in the lasso and elastic net regression are determined by a five-fold cross-validation scheme. In scenarios II to IV, data with missingness are generated after full omics data are simulated [16]. We evaluate NetMIM, NetMIM without MRF, FBM, NetMIM on complete data (NetMIM_CC), NetMIM without MRF prior on complete data (NetMIM_CC w/o MRF), and FBM on complete data (FBM_CC) using the simulated data with missingness. The methods on complete data mean that the methods are implemented only on the subjects with both gene expression and DNA methylation (remove the subjects with only one type of omics data).

For prior specification, the hyperparameters in the MRF prior are set as $d = -3$ and $f = 0.5$, which implies that the prior probability of a gene without neighborhoods is $\frac{\exp (-3)}{1+ \exp (-3)} \approx 0.05$, encouraging a sparse model. To avoid the phase transition problem, we prefer a small $f$ value and $0.5$ is also common in other methods adopting MRF prior in the variable selection [40]. For the scale parameters in the spike-and-slab prior, we set $\tau = \tau _{1}= \cdots = \tau _{K} = 1$ [41]. We refer to the sensitivity analysis results reported in the Supplementary Material for more details. As for the beta prior on the methylation probe selection, we set $a = 0.2$ and $b = 0.8$, indicating $20\%$ expected prior probability of inclusion. Vague priors are assigned for the variance in the model with $\delta _{1} = \delta _{2} = 0.001$.

### Results

**Figure 3:**
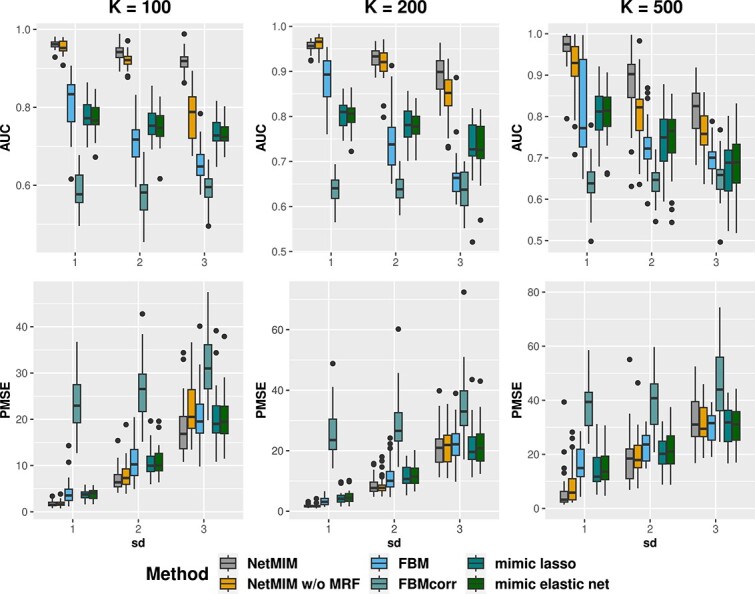
AUC and PMSE of different methods under different numbers of genes and model standard deviations, respectively; the number of genes $K$ varies from $100$, $200$, $500$; the x-axis represents the standard deviation $\sigma $ of the clinical model.

Figure 3 illustrates the AUC of gene selection results in the clinical model and the outcome prediction performance by PMSE on the validation dataset for scenario I. The proposed method, NetMIM, consistently outperforms the FBM method and the two other frequentist methods in variable selection and outcome prediction. Furthermore, the proposed method that incorporates structure information among genes achieves the best performance, better than NetMIM without MRF prior, especially for a higher number of genes. This observation suggests that incorporating extra structure information can enhance the model’s training. Figure 4 displays the posterior probability of gene inclusion for the three Bayesian methods, NetMIM, NetMIM without MRF, and FBM. When incorporating the network structure, the signals of effective genes are higher. For the FBM method, the average posterior probability of inclusion of $\boldsymbol{\gamma }^{\bar{M}}$ is higher than that of $\boldsymbol{\gamma }^{M}$, making it challenging to select $\boldsymbol{\gamma }^{M}$ and resulting in a lower AUC in feature selection. The reason for the phenomenon is that the estimated variance in the slab distribution for $\mathbf{E}^{\bar{M}}$ features was smaller than that for $\mathbf{E}^{M}$ features in the FBM model. Higher variances will introduce a greater penalty on the regression coefficients. As a result, they are more likely to shrink toward zero.

**Figure 5:**
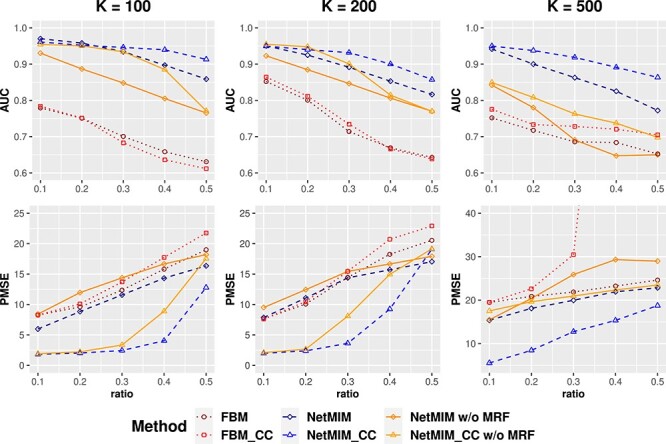
AUC and PMSE of different methods under different missing ratios for gene expression; the horizontal axis is the proportion of subjects without mRNA expression in the training data; the number of genes $K$ varies from $100$, $200$, $500$.

**Figure 6:**
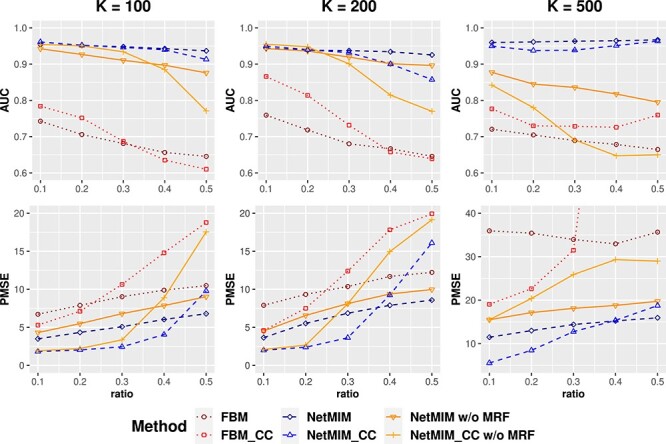
AUC and PMSE of different methods under different missing ratios for DNA methylation; the horizontal axis is the proportion of subjects without DNA methylation in the training data; the number of genes $K$ varies from $100$, $200$, $500$.

In scenario II, data with missingness are generated with different ratios of subjects whose gene expression is missing. Figure 5 displays the AUC of gene selection and outcome prediction in scenario II. In most cases, incorporating MRF prior improves the model performance, leading to better feature selection and model prediction (The blue line is better than the corresponding orange line). Furthermore, NetMIM_CC performs better than NetMIM with or without MRF prior. It is better to remove subjects without gene expression. The augmentation of gene expression introduces more uncertainty for feature selection and model prediction. In scenario III, data with missingness are generated with different ratios of subjects whose DNA methylation is missing. Figure 6 demonstrates the AUC of gene selection and outcome prediction in scenario III. Similarly, incorporating MRF prior leads to better feature selection and model prediction (The blue line is better than the corresponding orange line). When the missing ratio is small, the methods (both with MRF and without MRF) using complete data perform better, but the methods incorporating missing data perform better when the missing ratio is high. The influence of missing ratio on model performance is similar to a bivariate normal distribution, which we explored in Section 3 of the Supplementary Materials. In scenario IV, half of the total subjects are missing in gene expression or DNA methylation, with different proportions of subjects with missing gene expression. In this case, NetMIM performs the best, as shown in Figure S4. The AUCPRs of feature selection results in all simulations are shown in Figure S3 of Supplementary Materials, which also illustrate similar results as AUCs.

**Figure 7:**
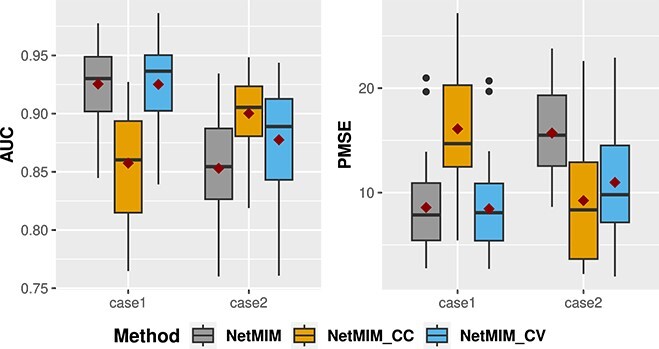
Boxplot of AUC and PMSE of different methods and CV scheme in two cases; the red point represents the mean value of the measure; Case 1 is $50\%$ missing ratio with $K=100$ in scenario III, and case 2 is $50\%$ missing ratio with $K=100$ in scenario II.

The question arises as to when we should include subjects without part of the omics data. Although we have explored the phenomenon that including subjects with missing data may sometimes decrease the model performance in the Supplementary Material, it is difficult to find an exact criterion to make a decision. Therefore, following the procedure in [16], we design a five-fold cross-validation scheme to determine whether to include those subjects or not. Specifically, we divide the training data into five equal parts with the same missing ratio. Each time we use one part as the validation set, and use the remaining as the training data set. NetMIM is trained on the whole subjects but NetMIM_CC is trained only on the subjects with complete data. The PMSE on the validation set for these two methods is computed from the subjects with complete data. The procedure is repeated across all five parts, and the method with the smaller average PMSE is selected as the final training method. Finally, the selected method is trained on the original training data set. This cross-validation scheme is denoted as NetMIM_CV. We also conduct some simulations for NetMIM, NetMIM_CC, and NetMIM_CV. Figure 7 shows that NetMIM_CV can achieve good performance in both cases where NetMIM performs better in case 1, but NetMIM_CC performs better in case 2 (case 1 is $50\%$ missing ratio with $K=100$ in scenario III, and case 2 is $50\%$ missing ratio with $K=100$ in scenario II).

## Evaluation on continuous response

The dataset is a public dataset (GSE65205) from a case-control study of atopic asthma and nasal epithelial DNA methylation in 72 predominantly African American children [42], with complete DNA methylation data from Illumina 450k chips and mRNA expression from Agilent-028004 SurePrint G3 Human GE 8x60K Microarray data. We used the M-value for methylation level for better model fitting. We are interested in the genes with methylation probes in the promoter regions and belonging to 20 pathways in the enrichment analysis of differentially expressed genes for another independent nasal epithelial data [43], resulting in 689 genes and 1844 methylation probes. To construct the interaction network $\mathbf{G}$, we set $g_{jl} = 1$ if there exists a direct interaction in a pathway for gene $j$ and gene $l$ and $g_{jl} = 0$ otherwise. Serum Immunoglobulin E (IgE) level is a primary clinical outcome in children’s asthma studies. We take log-transformed IgE level as our clinical response with age and gender as clinical variables. We consider five scenarios: (i) missing gene expression 20% (E:20%), (ii) missing gene expression 40% (E:40%), (iii) missing DNA methylation 20% (M:20%), (iv) missing DNA methylation 40% (M:40%), (v) no missing omics data (Full Set). We used 20 subjects as the test data in each scenario and repeated 10 times. The hyperparameters to control the MRF prior are set as $d = -4$ and $f = 0.5$ to assign a prior probability of inclusion of genes as approximately $0.02$. The other hyperparameters are the same as the simulation studies.

Table 1 shows the RMSE of outcome prediction of different methods on the test dataset in four scenarios. The lasso and elastic net method is the integrative analysis’s regression model concatenating mRNA expression and DNA methylation. NetMIM achieved the best performance under model prediction in the first three scenarios. Moreover, incorporating subjects with missing omics data improved model performance in NetMIM and FBM (NetMIM versus NetMIM_CC and FBM versus FBM_CC). The MRF prior also contributed to the improvement of model performance (NetMIM versus NetMIM w/o MRF and NetMIM_CC versus NetMIM_CC w/o MRF). Finally, it is worth noting that the mimic methods demonstrated better performance compared with the vanilla methods, particularly in the case of the elastic-net method, which illustrates the contributions of the mechanistic model.

**Table 1:** Means and standard deviations (in parentheses) of PMSE on a test dataset of different scenarios for the GSE65205 dataset; mimic lasso, mimic elastic net, lasso, and elastic net are implemented on the complete dataset; the results are based on 10 replications.

**Method**	**Scenario**				
	**(i) (E:20%)**	**(ii) (E:40%)**	**(iii) (M:20%)**	**(iv) (M:40%)**	**(v) (Full Set)**
NetMIM	**1.89 (0.50)**	**1.95 (0.45)**	**1.97 (0.46)**	2.11 (0.57)	**1.83 (0.52)**
FBM	2.38 (0.41)	2.52 (0.43)	2.34 (0.39)	2.51 (0.45)	2.24 (0.34)
NetMIM_CC	2.12 (0.59)	2.26 (0.65)	2.16 (0.54)	2.31 (0.71)	-
FBM_CC	2.44 (0.40)	2.67 (0.56)	2.35 (0.46)	3.01 (1.04)	-
NetMIM w/o MRF	1.94 (0.42)	2.01 (0.33)	2.02 (0.48)	**2.06 (0.60)**	1.91 (0.45)
NetMIM_CC w/o MRF	2.19 (0.61)	2.37 (0.75)	2.23 (0.64)	2.39 (0.87)	-
Mimic lasso	2.61 (0.79)	2.68 (0.51)	2.55 (0.98)	2.70 (0.76)	2.43 (0.44)
Mimic elastic net	2.05 (0.37)	2.18 (0.49)	2.15 (0.50)	2.19 (0.68)	2.01 (0.34)
Lasso	2.62 (0.69)	2.71 (0.50)	2.57 (0.46)	2.93 (0.74)	2.46 (0.51)
Elastic net	2.12 (0.39)	2.50 (0.55)	2.38 (0.60)	2.74 (0.73)	2.10 (0.44)

## Evaluation on survival response

In this section, we present an application of the proposed method to KIRC data from TCGA data portal, with DNA methylation data from Illumina 450K chips and RNA-seq gene expression data. The dataset includes $N = 532$ kidney cancer patients, all of whom have gene expression data, but there are only $N_{obs}^{M} = 318$ subjects whose DNA methylation is measured, implying that almost $42\%$ of the subjects do not have DNA methylation data. Regarding clinical variables, the survival time of patients is the response with age and gender as clinical covariates. For genetic features, we are interested in the genes belonging to $29$ KEGG pathways in the enrichment analysis of differential genes for renal clear cell carcinoma, resulting in 1772 genes [44,45]. The RNA-seq counts are transformed into continuous TPM (transcripts per million) values, and DNA methylation levels are represented by the *M*-value. We filter out genes with a mean expression level less than $10$ or a standard deviation less than $5$, resulting in $K = 814$ genes for the analysis [16]. In addition, we select the methylation probes mapped to each gene in the promoter region, obtaining $J = 6099$ methylation probes. These types of regulations, with regulators close to the target, are called *cis*-regulation, as opposed to *trans*-regulation, where the regulators are far from the target gene. Due to the smaller effective size of *trans*-acting variants, it is more efficient to detect *cis*-acting variants with a relatively small sample size. In our model formulation, the last component, MRF prior on selection indicators $\gamma $, requires the interaction network $\mathbf{G}$ among $K = 814$ genes, which are extracted from the KEGG database with the R package KEGGgraph [46]. If there exists a direct interaction in a pathway for gene $j$ and gene $l$, then $g_{jl} = 1$; $g_{jl} = 0$ otherwise. To compare the performance of the proposed method including subjects with missing methylation data and complete data, we randomly split the complete data into training data (220) and test data (93) after removing subjects with survival time less than 30 days. The censoring proportions in each subset are the same ($66\%$). With respect to prior specification, the hyperparameters to control the MRF prior are set as $d = -4$ and $f = 0.5$ to assign a prior probability of inclusion of genes as approximately $0.02$. The other hyperparameters are the same as the simulation studies.

### Results

To assess convergence, we ran four MCMC chains independently from different initial values with trace plots shown in Supplementary Material. We computed the pairwise Pearson correlation coefficients of the marginal posterior probability of inclusion for $\boldsymbol{\gamma }$ between different chains to check the consistency of selection results. The correlation coefficients of the posterior probability of inclusion ranged from $0.883$ to $0.893$ for $\boldsymbol{\gamma }$ and from $0.892$ to $0.915$ for $\mathbf{Z}$ among the four chains, demonstrating good convergence and consistent variable selection results of our model. Furthermore, we compared the performance of NetMIM, NetMIM_CC, and NetMIM_CV. To demonstrate the effect of MRF prior, we also implemented corresponding methods without MRF prior. As shown in Table 2, NetMIM performed best on the test dataset. The methods incorporating MRF prior achieved better model prediction performance. The FBM method is specifically tailored for continuous response variables and does not have a generalization for survival response. Therefore, we did not assess its performance on this particular real dataset for survival analysis. Instead, we compared NetMIM with recent survival analysis methods, including DeepOmix [47], BlockForest [48], and IPF_LASSO [49], whose configurations are detailed in the Supplementary Material. Regarding the efficiency, the computational time was $8.6$ h, $2.4$ h, $5.2$ min, and $2.3$ min for NetMIM, DeepOmix, BlockForest, and IPF_LASSO, respectively. NetMIN is time-consuming due to the computationally intensive MCMC algorithms and the complex mechanistic model, even though it was written by Rcpp to accelerate computation. All experiments were implemented on a high-performance computing server with E5 - 2643 v4 CPU (20 M cache, 3.40 GHz) with 256GB memory.

**Table 2:** Means and standard deviations (in parentheses) of C-indices of training data and test data of different methods on KIRC data; DeepOmix, BlockForest, and IPF_LASSO are implemented on the complete data set; the analysis is repeated 10 times.

**Method**	**Training data**	**Test data**
NetMIM	0.892 (0.036)	**0.724** (0.057)
NetMIM_CC	**0.963** (0.015)	0.694 (0.055)
NetMIM_CV	0.913 (0.054)	0.711 (0.051)
NetMIM w/o MRF	0.811 (0.019)	0.710 (0.056)
NetMIM_CC w/o MRF	0.863 (0.025)	0.670 (0.051)
NetMIM_CV w/o MRF	0.822 (0.036)	0.685 (0.063)
DeepOmix	0.863 (0.023)	0.714 (0.057)
BlockForest	0.697 (0.029)	0.653 (0.063)
IPF_LASSO	0.563 (0.019)	0.537 (0.054)

Applying the NetMIM model to the training data with missingness, we identified 128 genes with only *type $M$ effect*, 33 genes with only *type $\bar{M}$ effect*, and $14$ genes with both *type $M$ effect* and *type $\bar{M}$ effect* when utilizing the median probability model for selection (PPI cutoff = $0.5$). This result implies that the 14 genes have effects modulated by both methylation and other mechanisms. Additionally, we identified 78 genes that are significantly modulated by at least one methylation probe mapped to their promoter regions with a threshold equal to $0.5$. Among the effective methylation probes, $72\%$ of them were negatively associated with gene expression. These results illustrate the biological function of DNA methylation, which usually represses gene expression. As a comparison, the NetMIM without MRF model only identified two genes associated with survival time, as shown in Supplementary Figure S8.

### Biological findings

To validate our findings in biology, we investigated the gene symbols associated with survival time, particularly focusing on the 14 genes with both *type $M$ effect* and *type $\bar{M}$ effect*. Among the $28$ effects ($14$*type $M$ effects* and $14$*type $\bar{M}$ effects*), $75\%$ were negatively associated with the survival time response. Specifically, KRAS had the maximum absolute regression coefficient $\beta ^{\bar{M}} = -0.23$, which is a well-established tumor-driver gene involved in cancer initiation, development, and progression. MYC and THBS3 were also negatively related to survival time. MYC is an oncogene that is frequently amplified in cancer cells. For THBS3, recent studies have shown that the THBS family plays a crucial role in the development and progression of human cancer [50]. More details for other genes are shown in the Supplementary Material.

We next investigated whether the total $175$ identified genes represented better functional annotation in a biological sense by employing databases for annotation visualization and integrated discovery (DAVID) [51]. Annotation terms at a $0.05$ threshold applied to adjusted *P*-values were selected [52]. Under the KEGG pathway subcategory shown in Figure S11, the pathway Focal adhesion had a relatively large gene ratio and the smallest adjusted *P*-value ($1.5 \times 10^{-140}$). The terms PI3K-Akt signaling pathway and pathways in cancer followed, with the second and third smallest adjusted *P*-values ($7.8 \times 10^{-77}$ and $2.8 \times 10^{-73}$, respectively). Proteins in the Focal adhesion are associated with cancer metastasis, which is responsible for as many as $90\%$ of cancer-associated deaths in patients [53]. The PI3K-Akt signaling pathway is an intracellular signaling pathway that is important in regulating the cell cycle and is a central regulator pathway of several cancers, such as ovarian cancer and breast cancer. We comment on interesting findings from these pathways and other functional terms in the Supplementary Material.

In addition to our primary analysis, we have also applied NetMIM to the LUAD data obtained from TCGA data portal. Detailed information on this analysis can be found in the Supplementary Materials. In the LUAD analysis, we observed that the PI3K-Akt signaling pathway (*P*-value = $1.3 \times 10^{-45}$), Pathways in cancer (*P*-value = $5.9 \times 10^{-44}$), and Focal adhesion (*P*-value = $1.0 \times 10^{-43}$) were identified as the top three enriched pathways for the genes identified in the analysis. This finding further emphasizes the significance of these pathways in the development and progression of human cancers. The consistency of these enriched pathways across different datasets reinforces their importance and suggests their potential as therapeutic targets in cancer research.

## Conclusion and discussion

The heterogeneity and high variability of omics data pose challenges to integrative analysis. Incorporating structural dependencies among genes or other genetic features can improve the performance of integrative analysis. However, only a few existing Bayesian hierarchical models that investigate the association between genes and clinical outcomes consider the structural information among genetic features, especially the pathway information among genes. Another characteristic of multi-omics data integration is the potential for a large proportion of missing data since not all patients contain all omics data.

To address these challenges, we propose a network-based multi-omics integrative framework with missingness to perform biomarker selection, outcome prediction, and handling of missing data. Simulation studies have demonstrated that incorporating pathway information among genes improves the performance of feature selection and model prediction. Extensive simulations with missing data indicated that including subjects with incomplete omics data is not always favored over the complete case. To decide on the method of handling missing data, a cross-validation scheme was proposed, which achieved similar performance to the better one. Our proposed method provides a comprehensive approach to handle multi-omics data with missingness and incorporate pathway information among genes. It can improve the accuracy and interpretability of prognostic biomarker selection and analysis.

Despite its remarkable performance, our framework has some limitations. The MCMC algorithm has a high computation burden, even with optimized R code using C++. Additionally, our framework only includes two types of omics data (DNA methylation and mRNA expression), limiting its practical application. In the future, we will explore developing a framework that integrates more types of omics data, including single-nucleotide polymorphisms and micro RNA [36]. Furthermore, several extensions of our model are worth exploring. Firstly, considering the heterogeneity of cancers, we could extend the model to a finite mixture model, clustering patients to find cancer subtypes and estimating the number of clusters directly from the data. Secondly, we could not only propose a structural prior on the effects between DNA methylation and gene expression [54] but also consider the interaction effects of gene expression and DNA methylation on clinic outcomes. Finally, for the gene expression part regulated by other mechanisms, we could construct a model given other regulators’ measurements are known, such as pre-defined transcription factors.

**Key Points**

- The Dirac spike-and-slab feature selection priors are utilized to identify efficient biomarkers, achieving zero coefficient exactly.
- The integrative framework incorporates the known structural dependencies among biomarkers into feature selection via a Markov random field prior.
- Via data augmentation approaches, the integrative framework is generalized to different clinical outcomes, including continuous, binary, and right-censored responses.

## Supplementary Material

Supplymentary_material_bbae454

## Data Availability

The code and data are freely available on GitHub at https://github.com/new-zbc/NetMIM.

## References

[1] Mengyun W , Yi H, Ma S. Vertical integration methods for gene expression data analysis. *Brief Bioinform* 2021; 22. 10.1093/bib/bbaa169. PMC8138889 32793970

[2] Richardson S , Tseng GC, Sun W. Statistical methods in integrative genomics. *Annu Rev Stat Appl* 2016; 3:181–209. 10.1146/annurev-statistics-041715-033506. 27482531 PMC4963036

[3] Karczewski KJ , Snyder MP. Integrative omics for health and disease. *Nat Rev Genet* 2018; 19:299–310. 10.1038/nrg.2018.4. 29479082 PMC5990367

[4] Tseng GC , Ghosh D, Feingold E. Comprehensive literature review and statistical considerations for microarray meta-analysis. *Nucleic Acids Res* 2012; 40:3785–99. 10.1093/nar/gkr1265. 22262733 PMC3351145

[5] Shen R , Olshen AB, Ladanyi M. Integrative clustering of multiple genomic data types using a joint latent variable model with application to breast and lung cancer subtype analysis. *Bioinformatics* 2009; 25:2906–12. 10.1093/bioinformatics/btp543. 19759197 PMC2800366

[6] Kim S , Oesterreich S, Kim S. et al. Integrative clustering of multi-level omics data for disease subtype discovery using sequential double regularization. *Biostatistics* 2017; 18:165–79. 10.1093/biostatistics/kxw039. 27549122 PMC5255053

[7] Shen R , Wang S, Mo Q. Sparse integrative clustering of multiple omics data sets. *Ann Appl Stat* 2013; 7:269–94. 10.1214/12-AOAS578. 24587839 PMC3935438

[8] Lock EF , Dunson DB. Bayesian consensus clustering. *Bioinformatics* 2013; 29:2610–6. 10.1093/bioinformatics/btt425. 23990412 PMC3789539

[9] Swanson DM , Lien T, Bergholtz H. et al. A Bayesian two-way latent structure model for genomic data integration reveals few pan-genomic cluster subtypes in a breast cancer cohort. *Bioinformatics* 2019; 35:4886–97. 10.1093/bioinformatics/btz381. 31077301

[10] Gross SM , Tibshirani R. Collaborative regression. *Biostatistics* 2015; 16:326–38. 10.1093/biostatistics/kxu047. 25406332 PMC4441100

[11] Luo C , Liu J, Dey DK. et al. Canonical variate regression biostatistics. *Biostatistics* 2016; 17:468–83. 10.1093/biostatistics/kxw001. 26861909 PMC5006412

[12] Zhu R , Zhao Q, Zhao H. et al. Integrating multidimensional omics data for cancer outcome. *Biostatistics* 2016; 17:605–18. 10.1093/biostatistics/kxw010. 26980320 PMC5031941

[13] Jing X , Peng W, Chen Y. et al. A hierarchical integration deep flexible neural forest framework for cancer subtype classification by integrating multi-omics data. *BMC Bioinformatics* 2019; 20:1–11. 10.1186/s12859-019-3116-7. 31660856 PMC6819613

[14] Sun D , Wang M, Li A. A multimodal deep neural network for human breast cancer prognosis prediction by integrating multi-dimensional data. *IEEE/ACM Trans Comput Biol Bioinform* 2018; 16:841–50. 10.1109/TCBB.2018.2806438. 29994639

[15] Wang W , Baladandayuthapani V, Morris JS. et al. iBAG: Integrative Bayesian analysis of high-dimensional multiplatform genomics data. *Bioinformatics* 2013; 29:149–59. 10.1093/bioinformatics/bts655. 23142963 PMC3546799

[16] Fang Z , Ma T, Tang G. et al. Bayesian integrative model for multi-omics data with missingness. *Bioinformatics* 2018; 34:3801–8. 10.1093/bioinformatics/bty775. 30184058 PMC6223369

[17] Li Q , Cassese A, Guindani M. et al. Bayesian negative binomial mixture regression models for the analysis of sequence count and methylation data. *Biometrics* 2019; 75:183–92. 10.1111/biom.12962. 30125947

[18] Meng C , Zeleznik OA, Thallinger GG. et al. Dimension reduction techniques for the integrative analysis of multi-omics data. *Brief Bioinform* 2016; 17:628–41. 10.1093/bib/bbv108. 26969681 PMC4945831

[19] Tibshirani R . Regression shrinkage and selection via the lasso. *J R Stat Soc B Methodol* 1996; 58:267–88. 10.1111/j.2517-6161.1996.tb02080.x.

[20] Zou H , Hastie T. Regularization and variable selection via the elastic net. *J R Stat Soc Series B Stat Methodology* 2005; 67:301–20. 10.1111/j.1467-9868.2005.00503.x.

[21] Tibshirani R , Saunders M, Rosset S. et al. Sparsity and smoothness via the fused lasso. *J R Stat Soc Series B Stat Methodology* 2005; 67:91–108. 10.1111/j.1467-9868.2005.00490.x.

[22] Park T , Casella G. The Bayesian lasso. *J Am Stat Assoc* 2008; 103:681–6. 10.1198/016214508000000337.

[23] Ročková V , George EI. The spike-and-slab lasso. *J Am Stat Assoc* 2018; 113:431–44. 10.1080/01621459.2016.1260469.

[24] Biswas N , Mackey L, Meng X-L. Scalable spike-and-slab. In: Proceedings of the 39th International Conference on Machine Learning 2022;162:2021–40.

[25] Herrmann M , Probst P, Hornung R. et al. Large-scale benchmark study of survival prediction methods using multi-omics data. *Brief Bioinform* 2021;22:1–15. 10.1093/bib/bbaa167. PMC8138887 32823283

[26] Cantini L , Zakeri P, Hernandez C. et al. Benchmarking joint multi-omics dimensionality reduction approaches for the study of cancer. *Nat Commun* 2021; 12:124. 10.1038/s41467-020-20430-7. 33402734 PMC7785750

[27] Wissel D , Rowson D, Boeva V. Systematic comparison of multi-omics survival models reveals a widespread lack of noise resistance. *Cell Reports Methods* 2023; 3:100461. 10.1016/j.crmeth.2023.100461. 37159669 PMC10162996

[28] Bin Gao X , Liu HL, Cui Y. Integrative analysis of genetical genomics data incorporating network structures. *Biometrics* 2019; 75:1063–75. 10.1111/biom.13072. 31009063 PMC6810723

[29] Stingo FC , Vannucci M. Variable selection for discriminant analysis with Markov random field priors for the analysis of microarray data. *Bioinformatics* 2011; 27:495–501. 10.1093/bioinformatics/btq690. 21159623 PMC3105481

[30] Li Y , Shaodong X, Ma S. et al. Network-based cancer heterogeneity analysis incorporating multi-view of prior information. *Bioinformatics* 2022; 38:2855–62. 10.1093/bioinformatics/btac183. 35561185 PMC9113254

[31] Van De Velden M , Bijmolt THA. Generalized canonical correlation analysis of matrices with missing rows: A simulation study. *Psychometrika* 2006; 71:323–31. 10.1007/s11336-004-1168-9. 28197957

[32] Voillet V , Besse P, Liaubet L. et al. Handling missing rows in multi-omics data integration: Multiple imputation in multiple factor analysis framework. *BMC Bioinformatics* 2016; 17:1–16. 27716030 10.1186/s12859-016-1273-5 PMC5048483

[33] Daniels MJ , Wang C, Marcus BH. Fully Bayesian inference under ignorable missingness in the presence of auxiliary covariates. *Biometrics* 2014; 70:62–72. 10.1111/biom.12121. 24571539 PMC4007313

[34] Das S , Chen M-H, Kim S. et al. A Bayesian structural equations model for multilevel data with missing responses and missing covariates. *Bayesian Anal* 2008; 3:197–224. 10.1214/08-BA308.

[35] Erler NS , Rizopoulos D, van Rosmalen J. et al. Dealing with missing covariates in epidemiologic studies: A comparison between multiple imputation and a full Bayesian approach. *Stat Med* 2016; 35:2955–74. 10.1002/sim.6944. 27042954

[36] Chekouo T , Stingo FC, Doecke JD. et al. miRNA–target gene regulatory networks: A Bayesian integrative approach to biomarker selection with application to kidney cancer. *Biometrics* 2015; 71:428–38. 10.1111/biom.12266. 25639276 PMC4499566

[37] Newton MA , Noueiry A, Sarkar D. et al. Detecting differential gene expression with a semiparametric hierarchical mixture method. *Biostatistics* 2004; 5:155–76. 10.1093/biostatistics/5.2.155. 15054023

[38] Peterson C , Stingo FC, Vannucci M. Bayesian inference of multiple gaussian graphical models. *J Am Stat Assoc* 2015; 110:159–74. 10.1080/01621459.2014.896806. 26078481 PMC4465207

[39] Harrell FE , Califf RM, Pryor DB. et al. Evaluating the yield of medical tests. *JAMA* 1982; 247:2543–6. 10.1001/jama.1982.03320430047030. 7069920

[40] Cao X , Lee K. Joint Bayesian variable and DAG selection consistency for high-dimensional regression models with network-structured covariates. Stat Sin 2021;31:1509–30. 10.5705/ss.202019.0202.

[41] Sha N , Vannucci M, Tadesse MG. et al. Bayesian variable selection in multinomial probit models to identify molecular signatures of disease stage. *Biometrics* 2004; 60:812–9. 10.1111/j.0006-341X.2004.00233.x. 15339306

[42] Yang IV , Pedersen BS, Liu AH. et al. The nasal methylome and childhood atopic asthma. *J Allergy Clin Immunol* 2017; 139:1478–88. 10.1016/j.jaci.2016.07.036. 27745942 PMC5391298

[43] Forno E , Wang T, Qi C. et al. DNA methylation in nasal epithelium, atopy, and atopic asthma in children: A genome-wide study. *The lancet*. *Respir Med* 2019; 7:336–46. 10.1016/S2213-2600(18)30466-1. PMC6441380 30584054

[44] Yang W , Yoshigoe K, Qin X. et al. Identification of genes and pathways involved in kidney renal clear cell carcinoma. *BMC Bioinformatics* 2014; 15:1–10. 10.1186/1471-2105-15-S17-S2. 25559354 PMC4304191

[45] Yuan L , Zeng G, Chen L. et al. Identification of key genes and pathways in human clear cell renal cell carcinoma (ccRCC) by co-expression analysis. *Int J Biol Sci* 2018; 14:266–79. 10.7150/ijbs.23574. 29559845 PMC5859473

[46] Zhang JD , Wiemann S. KEGGgraph: A graph approach to KEGG PATHWAY in R and bioconductor. *Bioinformatics* 2009; 25:1470–1. 19307239 10.1093/bioinformatics/btp167 PMC2682514

[47] Zhao L , Dong Q, Luo C. et al. Deepomix: A scalable and interpretable multi-omics deep learning framework and application in cancer survival analysis. *Comput Struct Biotechnol J* 2021; 19:2719–25. 10.1016/j.csbj.2021.04.067. 34093987 PMC8131983

[48] Hornung R , Wright MN. Block forests: Random forests for blocks of clinical and omics covariate data. *BMC Bioinformatics* 2019; 20:1–17. 31248362 10.1186/s12859-019-2942-y PMC6598279

[49] Boulesteix A-L , De Bin R, Jiang X. et al. IPF-LASSO: Integrative-penalized regression with penalty factors for prediction based on multi-omics data. *Comput Math Methods Med* 2017; 2017:1–14. 10.1155/2017/7691937. PMC5435977 28546826

[50] Zhang C , Chenyu H, Kunqi S. et al. The integrative analysis of thrombospondin family genes in pan-cancer reveals that THBS2 facilitates gastrointestinal cancer metastasis. *J Oncol* 2021; 2021:1–19. 10.1155/2021/4405491. PMC8598331 34804159

[51] Dennis G , ShermanBT, HosackDA. et al. DAVID: Database for annotation, visualization, and integrated discovery. *Genome Biol*2003; 4:1–11.12734009

[52] Benjamini Y , Hochberg Y. Controlling the false discovery rate: A practical and powerful approach to multiple testing. *J R Stat Soc B Methodol* 1995; 57:289–300. 10.1111/j.2517-6161.1995.tb02031.x.

[53] Gorka J , Marona P, Kwapisz O. et al. MCPIP1 regulates focal adhesion kinase and rho GTPase-dependent migration in clear cell renal cell carcinoma. *Eur J Pharmacol* 2022; 922:174804. 10.1016/j.ejphar.2022.174804. 35257717

[54] Cassese A , Guindani M, Tadesse MG. et al. A hierarchical Bayesian model for inference of copy number variants and their association to gene expression. *Ann Appl Stat* 2014;8:148–75. 10.1214/13-AOAS705. PMC4018204 24834139

