# Supplementary material for: NetMIM: network-based multi-omics integration with block missingness for biomarker selection and disease outcome prediction
Source: Brief Bioinform. 2024 Sep 17;25(5):bbae454. doi: 10.1093/bib/bbae454 (PMC11407451; doi:10.1093/bib/bbae454)
Supplement: Supplymentary_material_bbae454 [file supplymentary_material_bbae454.pdf]

Supplementary materials for Network-based multi-omics integration with block missingness for biomarker selection and disease outcome prediction

By Bencong Zhu, Zhen Zhang, Suet Yi Leung and Xiaodan Fan

August 30, 2024

1 Full MCMC procedure

We use $D = \{\boldsymbol{Y}_N, \mathbf{M}_{N\times J}, \mathbf{E}_{N\times K}, \mathbf{C}_{N\times L}\}$ to represent full data set, including observed data and missing data, where $\mathbf{M}_{N\times J} = (\mathbf{M}^T_{N^M_{obs}\times J}, \mathbf{M}^T_{N^M_{mis}\times J})^T$ and $\mathbf{E}_{N\times K} = (\mathbf{E}^T_{N^E_{obs}\times K}, \mathbf{E}^T_{N^E_{mis}\times K})^T$. The model parameters is denoted by $\Theta = \{\boldsymbol{\beta}^C, \boldsymbol{\beta}^M, \boldsymbol{\beta}^{\bar{M}}, \boldsymbol{\gamma}^M, \boldsymbol{\gamma}^{\bar{M}}, \Omega, \mathbf{Z}, \sigma^2, \sigma^2_{1:K}\}$. The full Bayesian model can be written as

$$P(\Theta \mid D) \propto P(D \mid \Theta)P(\Theta)$$

where $P(D \mid \Theta)$ is the data likelihood and $P(\Theta)$ is the prior distribution of parameters. The above formula can be written out in detail as

$$\begin{aligned} P(\Theta \mid D) \propto\ & P(\boldsymbol{Y}_N \mid \mathbf{M}_{N\times J}, \mathbf{E}_{N\times K}, \mathbf{C}_{N\times L}, \Omega, \boldsymbol{\beta}^C, \boldsymbol{\beta}^M, \boldsymbol{\beta}^{\bar{M}}, \sigma^2) \times P(\mathbf{E}_{N\times K} \mid \mathbf{M}_{N\times J}, \Omega, \sigma^2_{1:K}) \\ & \times P(\boldsymbol{\beta}^C) \times P(\boldsymbol{\beta}^M \mid \boldsymbol{\gamma}^M) \times P(\boldsymbol{\beta}^{\bar{M}} \mid \boldsymbol{\gamma}^{\bar{M}}) \times P(\boldsymbol{\gamma}^M, \boldsymbol{\gamma}^{\bar{M}} \mid G) \times P(\Omega \mid \mathbf{Z}) \times P(\mathbf{Z} \mid \pi_{1:K}) \\ & \times P(\pi_{1:K}) \times P(\sigma^2) \times P(\sigma^2_{1:K}) \end{aligned}$$

1.1 Updating parameters in the clinical model

Given Ω, sample $\boldsymbol{\gamma}^{\bar{M}}$ and $\boldsymbol{\gamma}^{M}$ via a Metropolis step. The posterior conditional distribution is

$$
\begin{aligned}
& P(\boldsymbol{\beta}^C, \boldsymbol{\beta}^M, \boldsymbol{\beta}^{\bar{M}}, \boldsymbol{\gamma}^M, \boldsymbol{\gamma}^{\bar{M}}, \sigma^2 \mid D, \Omega) \\
& \propto P(\boldsymbol{Y} \mid \mathbf{M}, \mathbf{E}, \mathbf{C}, \Omega, \boldsymbol{\beta}^C, \boldsymbol{\beta}^M, \boldsymbol{\beta}^{\bar{M}}, \sigma^2) \times P(\boldsymbol{\beta}^C) \times P(\boldsymbol{\beta}^M \mid \boldsymbol{\gamma}^M) \\
& \times P(\boldsymbol{\beta}^{\bar{M}} \mid \boldsymbol{\gamma}^{\bar{M}}) \times P(\boldsymbol{\gamma}^M, \boldsymbol{\gamma}^{\bar{M}} \mid G) \times P(\sigma^2)
\end{aligned}
$$

We can integrate out the nuisance parameters $(\boldsymbol{\beta}^C, \boldsymbol{\beta}^M, \boldsymbol{\beta}^{\bar{M}}, \sigma^2)$, obtaining the posterior

$$
P(\boldsymbol{\gamma}^M, \boldsymbol{\gamma}^{\bar{M}} \mid D, \Omega) \propto P(\boldsymbol{Y} \mid \mathbf{M}, \mathbf{E}, \mathbf{C}, \Omega, \boldsymbol{\gamma}^M, \boldsymbol{\gamma}^{\bar{M}}, \tau_c, \tau, \delta_1, \delta_2) \times P(\boldsymbol{\gamma}^M, \boldsymbol{\gamma}^{\bar{M}} \mid G, d, f)
$$

where $(\tau_c, \tau, \delta_1, \delta_2, d, f)$ are the hyperparameters in the model and G is the prior network information among the genes.

Denoting $\mathbf{X}_{N\times 2K} = (\mathbf{E} - \mathbf{M}\Omega, \mathbf{M}\Omega)$, $\boldsymbol{\beta} = \left((\boldsymbol{\beta}^{\bar{M}})^T, (\boldsymbol{\beta}^M)^T\right)^T$ and $\boldsymbol{\gamma} = \left((\boldsymbol{\gamma}^{\bar{M}})^T, (\boldsymbol{\gamma}^M)^T\right)^T$, the integrated likelihood $P(\boldsymbol{Y} \mid \mathbf{M}, \mathbf{E}, \mathbf{C}, \Omega, \boldsymbol{\gamma}^M, \boldsymbol{\gamma}^{\bar{M}}) = P(\boldsymbol{Y} \mid \mathbf{C}, \mathbf{X}, \boldsymbol{\gamma})$ where we omit the hyperparameters.
We adopt a stochastic search procedure to sample indicators $\boldsymbol{\gamma}$. Given the indicators $\boldsymbol{\gamma}^{\text{old}}$, we choose a move between Add/Delete move and Swap with probability ρ. The Add/Delete move randomly selects one of the elements in $\boldsymbol{\gamma}^{\text{old}}$ and change its value (form 0 to 1 or from 1 to 0). The Swap move randomly selects two elements in $\boldsymbol{\gamma}^{\text{old}}$ with different inclusion statuses and swaps their values. A new candidate $\boldsymbol{\gamma}^{\text{new}}$ is accepted with probability

$$
\min\left\{\frac{P\left(\boldsymbol{Y} \mid \mathbf{C}, \mathbf{X}, \boldsymbol{\gamma}^{\text{new}}\right) P\left(\boldsymbol{\gamma}^{\text{new}} \mid G\right)}{P\left(\boldsymbol{Y} \mid \mathbf{C}, \mathbf{X}, \boldsymbol{\gamma}^{\text{old}}\right) P\left(\boldsymbol{\gamma}^{\text{old}} \mid G\right)}, 1\right\}
$$

The formula of $P(\boldsymbol{Y} \mid \mathbf{C}, \mathbf{X}, \boldsymbol{\gamma})$ is obtained by integration

$$
\begin{aligned}
&P(\boldsymbol{Y} \mid \mathbf{C}, \mathbf{X}, \boldsymbol{\gamma}) \\
&= \int p(\boldsymbol{Y}, \boldsymbol{\beta}^c, \boldsymbol{\beta}, \sigma^2 \mid \mathbf{X}, \boldsymbol{\gamma}) d\boldsymbol{\beta}^c d\boldsymbol{\beta} d(\sigma^2) \\
&= \int p(\boldsymbol{Y} \mid \mathbf{C}, \mathbf{X}, \boldsymbol{\beta}^c, \boldsymbol{\beta}, \sigma^2) p(\boldsymbol{\beta}^c) p(\boldsymbol{\beta} \mid \boldsymbol{\gamma}) d\boldsymbol{\beta}^c d\boldsymbol{\beta} dP(\sigma^2) \\
&= \int \left(C_1 (2\pi)^{-L/2} \sigma^{-(N+L)} \exp\left\{ -\frac{||\boldsymbol{Y} - \mathbf{C}\boldsymbol{\beta}^c - \mathbf{X}\boldsymbol{\beta}||^2}{2\sigma^2} - \frac{\tau_c ||\boldsymbol{\beta}^c||^2}{2\sigma^2} \right\} d\boldsymbol{\beta}^c \right) p(\boldsymbol{\beta} \mid \boldsymbol{\gamma}) d\boldsymbol{\beta} dP(\sigma^2) \\
&= \int \left(C_1 \sigma^{-N} |\mathbf{U}_c|^{-1/2} \exp\left\{ -\frac{(\boldsymbol{Y} - \mathbf{X}_{\boldsymbol{\gamma}}\boldsymbol{\beta}_{\boldsymbol{\gamma}})^T (\mathbf{I} - \mathbf{H}_c)(\boldsymbol{Y} - \mathbf{X}_{\boldsymbol{\gamma}}\boldsymbol{\beta}_{\boldsymbol{\gamma}})}{2\sigma^2} \right\} p(\boldsymbol{\beta}_{\boldsymbol{\gamma}} \mid \boldsymbol{\gamma}) d\boldsymbol{\beta}_{\boldsymbol{\gamma}} \right) dP(\sigma^2) \\
&= \int \left(C_2 \tau^{|\gamma|/2} (2\pi)^{-|\gamma|/2} \sigma^{-(N+|\gamma|)} \exp\left\{ -\frac{(\boldsymbol{Y} - \mathbf{X}_{\boldsymbol{\gamma}}\boldsymbol{\beta}_{\boldsymbol{\gamma}})^T (\mathbf{I} - \mathbf{H}_c)(\boldsymbol{Y} - \mathbf{X}_{\boldsymbol{\gamma}}\boldsymbol{\beta}_{\boldsymbol{\gamma}}) + c||\boldsymbol{\beta}_{\boldsymbol{\gamma}}||^2}{2\sigma^2} \right\} d\boldsymbol{\beta}_{\boldsymbol{\gamma}} \right) dP(\sigma^2) \\
&= \int C_2 \tau^{k/2} \sigma^{-N} |\mathbf{U}_x|^{-1/2} \exp\left\{ -\frac{\boldsymbol{Y}^T (\mathbf{I} - \mathbf{H}_c) \boldsymbol{Y} - \boldsymbol{Y}^T (\mathbf{I} - \mathbf{H}_c) \mathbf{X}_{\boldsymbol{\gamma}} \mathbf{U}_x^{-1} \mathbf{X}_{\boldsymbol{\gamma}}^T (\mathbf{I} - \mathbf{H}_c) \mathbf{Y}}{2\sigma^2} \right\} p(\sigma^2) d(\sigma^2) \\
&= C_2 \tau^{|\gamma|/2} |\mathbf{U}_x|^{-1/2} \int \sigma^{-N} \exp(-Q/2\sigma^2) \frac{\delta_2^{\delta_1} \sigma^{-2(\delta_1 - 1)} \exp(-\delta_2/\sigma^2)}{\Gamma(\delta_1)} d(\sigma^2) \\
&= \frac{\tau_c^{L/2} \tau^{|\gamma|/2} \Gamma(N/2 + \delta_1) \delta_2^{\delta_1}}{(2\pi)^{N/2} |\mathbf{U}_c|^{1/2} |\mathbf{U}_x|^{1/2} \Gamma(\delta_1) (\delta_2 + Q/2)^{(N/2 + \delta_1)}} \\
&\propto \frac{\tau^{|\gamma|/2} \Gamma(N/2 + \delta_1)}{|\mathbf{U}_x|^{1/2} (\delta_2 + Q/2)^{(N/2 + \delta_1)}}
\end{aligned}
$$

where $\mathbf{U}_c = \mathbf{C}^T\mathbf{C} + \tau_c \mathbf{I}_L$, $\mathbf{H}_c = \mathbf{C}\mathbf{U}_x^{-1}\mathbf{C}^T$ and $\mathbf{U}_x = \mathbf{X}_{\boldsymbol{\gamma}}^T (\mathbf{I}_N - \mathbf{H}_c) \mathbf{X}_{\boldsymbol{\gamma}} + \tau \mathbf{I}_k$. The number Q is $Q = \boldsymbol{Y}^T(\mathbf{I}_N - \mathbf{H}_c)\boldsymbol{Y} - \boldsymbol{Y}^T(\mathbf{I}_N - \mathbf{H}_c)\mathbf{X}_{\boldsymbol{\gamma}}\mathbf{U}_x^{-1}\mathbf{X}_{\boldsymbol{\gamma}}^T(\mathbf{I}_N - \mathbf{H}_c)\mathbf{Y}$. The notation $\mathbf{X}_{\boldsymbol{\gamma}}$ represents the matrix keeping all the columns selected by the indicators $\boldsymbol{\gamma}$. The notation $|\gamma| = \sum_{k=1}^K \gamma_k^{\bar{M}} + \gamma_k^M$.

Given $(\Omega, \boldsymbol{\gamma}^{\bar{M}}, \boldsymbol{\gamma}^M, \sigma^2)$, sample $(\boldsymbol{\beta}^C, \boldsymbol{\beta}^{\bar{M}}, \boldsymbol{\beta}^M)$. If $\gamma_k^{\bar{M}} = 0$ or $\gamma_k^M = 0$, then $\beta_k^{\bar{M}} = 0$ or $\beta_k^M = 0$ correspondingly. We define $\boldsymbol{\beta}_{\boldsymbol{\gamma}}^{\bar{M}} = \{\beta_k^{\bar{M}} : \gamma_k^{\bar{M}} \neq 0, \text{ for } k = 1, \ldots, K\}$ and $\boldsymbol{\beta}_{\boldsymbol{\gamma}}^M = \{\beta_k^M : \gamma_k^M \neq 0, \text{ for } k = 1, \ldots, K\}$ as the nonzero coefficients. The row vector of nonzero coefficients $(\boldsymbol{\beta}^C, \boldsymbol{\beta}_{\boldsymbol{\gamma}}^{\bar{M}}, \boldsymbol{\beta}_{\boldsymbol{\gamma}}^M)$ are sampled from

$$
(\boldsymbol{\beta}^C, \boldsymbol{\beta}_{\boldsymbol{\gamma}}^{\bar{M}}, \boldsymbol{\beta}_{\boldsymbol{\gamma}}^M) \sim N(\Sigma^{-1}\mathbf{X}^T\boldsymbol{Y}, \sigma^2\Sigma)
$$

where $\mathbf{X} = (\mathbf{C}, \mathbf{X}_\gamma)$ and $\Sigma = (\mathbf{X}^T\mathbf{X} + diag\{\tau_c \mathbf{1}_L, \tau \mathbf{1}_{|\gamma|}\})^{-1}$. The notation $|\gamma| = \sum_{k=1}^K \gamma_k^{\bar{M}} + \gamma_k^M$.

Given $(\Omega, \boldsymbol{\beta}^C, \boldsymbol{\beta}^M, \boldsymbol{\beta}^{\bar{M}})$, sample σ^2 from an inverse gamma distribution.

$$\frac{1}{\sigma^2} \sim Gamma(N/2 + \delta_1, \frac{1}{2}\boldsymbol{\epsilon}^T\boldsymbol{\epsilon} + \delta_2)$$

where $\boldsymbol{\epsilon} = \boldsymbol{Y} - \mathbf{C}\boldsymbol{\beta}^C - (\mathbf{E} - \mathbf{M}\Omega)\boldsymbol{\beta}^{\bar{M}} - \mathbf{M}\Omega\boldsymbol{\beta}^M$.

1.2 Updating the parameters in the mechanistic model

For each gene $k = 1, \ldots, K$, we update the parameters iteratively.

Given $(\Omega_{-k}, \boldsymbol{\beta}^C, \boldsymbol{\beta}^M, \boldsymbol{\beta}^{\bar{M}}, \sigma^2, \sigma_k^2)$, update $\mathbf{Z}_k$ via a Metropolis step. The posterior conditional distribution is given by

$$P(\Omega_{\mathcal{J}_k,k}, \mathbf{Z}_{\mathcal{J}_k,k} \mid \Omega_{-k}, \boldsymbol{\beta}^C, \boldsymbol{\beta}^M, \boldsymbol{\beta}^{\bar{M}}, \sigma^2, \sigma_k^2, D)$$
$$\propto P(\boldsymbol{Y} \mid \mathbf{M}, \mathbf{E}, \mathbf{C}, \Omega, \boldsymbol{\beta}^C, \boldsymbol{\beta}^M, \boldsymbol{\beta}^{\bar{M}}, \sigma^2) \times P(\boldsymbol{E}_k \mid \mathbf{M}, \Omega_k, \sigma_k^2) \times P(\Omega_{\mathcal{J}_k,k} | \mathbf{Z}_{\mathcal{J}_k,k}, \sigma_k^2) \times P(\mathbf{Z}_{\mathcal{J}_k,k} \mid \pi_k) \times P(\pi_k)$$

where $\mathcal{J}_k$ represents the set of methylation probes mapped to the gene k, Ω_k is the k-th column of Ω, and $\Omega_{\mathcal{J}_k,k}$ are the coefficients of mapped methylation probs for gene k. Under the constraint that methylation probes in the promoter region of one gene only regulate the expression of that gene, we have $\Omega_{-\mathcal{J}_k,k} = 0$. In the prior distribution of $\mathbf{Z}_{\mathcal{J}_k,k}$, we can integrate out π_k, resulting in $P(\mathbf{Z}_{\mathcal{J}_k,k}) = \frac{B(a+|\mathbf{Z}_{\mathcal{J}_k,k}|, \quad b+|\mathcal{J}_k|-|\mathbf{Z}_{\mathcal{J}_k,k}|)}{B(a,b)}$, where $|\mathcal{J}_k|$ is the cardinality of the set, $|\mathbf{Z}_{\mathcal{J}_k,k}|$ is number of nonzero component in the indicators and $B(\cdot, \cdot)$ is beta function. Similarly, we integrate the regression parameters $\Omega_{\mathcal{J}_k,k}$ in the posterior conditional distribution.

$$P(\mathbf{Z}_{\mathcal{J}_k,k} \mid \Omega_{-k}, \boldsymbol{\beta}^C, \boldsymbol{\beta}^M, \boldsymbol{\beta}^{\bar{M}}, \sigma^2, \sigma_k^2, D) \propto f(\mathbf{Z}_{\mathcal{J}_k,k}, \Omega_{-k}, \boldsymbol{\beta}^C, \boldsymbol{\beta}^M, \boldsymbol{\beta}^{\bar{M}}, \sigma^2, \sigma_k^2, D) \times P(\mathbf{Z}_{\mathcal{J}_k,k})$$

In the formula,

$$f(\mathbf{Z}_{\mathcal{J}_k,k}, \Omega_{-k}, \boldsymbol{\beta}^C, \boldsymbol{\beta}^M, \boldsymbol{\beta}^{\bar{M}}, \sigma^2, \sigma_k^2, D)$$
$$= \int P(\boldsymbol{Y} \mid \mathbf{M}, \mathbf{E}, \mathbf{C}, \Omega_k, \Omega_{-k}, \boldsymbol{\beta}^C, \boldsymbol{\beta}^M, \boldsymbol{\beta}^{\bar{M}}, \sigma^2) \times P(\boldsymbol{E}_k \mid \mathbf{M}, \Omega_k, \sigma_k^2) \times P(\Omega_{\mathcal{J}_k,k} | \mathbf{Z}_{\mathcal{J}_k,k}, \sigma_k^2) d(\Omega_{\mathcal{J}_k,k})$$
$$= (2\pi)^{-N} \tau_k^{|\mathbf{Z}_{\mathcal{J}_k,k}|/2} \sigma^{-N} \sigma_k^{-N} |\mathbf{U}_k|^{-1/2} \exp\left\{ -\frac{\rho_k \bar{\boldsymbol{Y}}^T \bar{\boldsymbol{Y}} + \boldsymbol{E}_k^T \boldsymbol{E}_k - (\boldsymbol{E}_k^*)^T \mathbf{M}_{\mathbf{Z}_{\mathcal{J}_k,k}} \mathbf{U}_k^{-1} \mathbf{M}_{\mathbf{Z}_{\mathcal{J}_k,k}}^T \boldsymbol{E}_k^*}{2\sigma_k^2} \right\}$$

where $\rho_k = \sigma_k^2/\sigma^2$ and $\bar{\boldsymbol{Y}} = \boldsymbol{Y} - \mathbf{C}\boldsymbol{\beta}^C - \mathbf{E}\boldsymbol{\beta}^{\bar{M}} + \mathbf{M}\Omega_{-k}(\boldsymbol{\beta}_{-k}^{\bar{M}} - \boldsymbol{\beta}_{-k}^{M})$, $\quad \boldsymbol{E}_k^* = \rho_k * (\beta_k^{\bar{M}} - \beta_k^M) * \bar{\boldsymbol{Y}} + \boldsymbol{E}_k$. The matrix $\mathbf{U}_k$ is $\mathbf{U}_k = (\rho_k * (\beta_k^{\bar{M}} - \beta_k^M)^2 + 1)\mathbf{M}_{\mathbf{Z}_{\mathcal{J}_k,k}}^T \mathbf{M}_{\mathbf{Z}_{\mathcal{J}_k,k}} + \tau_k \mathbf{I}_{|\mathbf{Z}_{\mathcal{J}_k,k}|}$.

A similar stochastic search method is utilized to sample $\mathbf{Z}_{\mathcal{J}_k,k}$. The new candidate $\mathbf{Z}_{\mathcal{J}_k,k}^{\text{new}}$ is accepted with probability

$$\min\left\{\frac{P(\boldsymbol{Z}_{\mathcal{J}_k,k}^{\text{new}} \mid \Omega_{-k}, \boldsymbol{\beta}^C, \boldsymbol{\beta}^M, \boldsymbol{\beta}^{\bar{M}}, \sigma^2, \sigma_k^2, D)}{P(\boldsymbol{Z}_{\mathcal{J}_k,k}^{\text{old}} \mid \Omega_{-k}, \boldsymbol{\beta}^C, \boldsymbol{\beta}^M, \boldsymbol{\beta}^{\bar{M}}, \sigma^2, \sigma_k^2, D)}, 1\right\}$$

Given $(\boldsymbol{Z}_{\mathcal{J}_k,k}, \Omega_{-k}, \boldsymbol{\beta}^C, \boldsymbol{\beta}^M, \boldsymbol{\beta}^{\bar{M}}, \sigma^2, \sigma_k^2)$, sample $\Omega_{\mathcal{J}_k,k}$. If $z_{jk} = 0$, then $w_{jk} = 0$ for $j \in \mathcal{J}_k$. Let $\Omega_{\boldsymbol{Z}_{\mathcal{J}_k}}$ be the nonzero effect of methylation on gene k, we have

$$\Omega_{\mathbf{Z}_{\mathcal{J}_k}} \sim N(\mathbf{U}_k^{-1}\mathbf{M}_{\mathbf{Z}_{\mathcal{J}_k,k}}^T \boldsymbol{E}_k^*, \sigma_k^2 \mathbf{U}_k^{-1})$$

where $\mathbf{U}_k$ and $\boldsymbol{E}_k^*$ are the same as that in the aforementioned step.

Given Ω_k, sample σ_k^2 from an inverse gamma distribution.

$$\frac{1}{\sigma_k^2} \sim Gamma(N/2 + \delta_1, \frac{1}{2}\boldsymbol{\epsilon}_k^T \boldsymbol{\epsilon}_k + \delta_2)$$

where $\boldsymbol{\epsilon}_k = \boldsymbol{E}_k - \mathbf{M}\Omega_k$.

1.3 Updating missing data in omics data

If there are some samples not containing all types of omics data, we are required to update the missing omics data according to the previous model.

- Samples with missing gene expression are imputed as

$$\boldsymbol{E}_{N_{mis}^G,k} \sim N\left((\sigma^{-2}(\beta_k^{\bar{M}})^2 + \sigma^{-2})^{-1}(\sigma^{-2}A\beta_k^{\bar{M}} + \sigma_k^{-2}B), (\sigma^{-2}(\beta_k^{\bar{M}})^2 + \sigma^{-2})^{-1}\right)$$

  where $A = \boldsymbol{Y}_{N_{mis}^G} - \mathbf{C}_{N_{mis}^G}\boldsymbol{\beta}^C - \mathbf{M}_{N_{mis}^G}\Omega(\boldsymbol{\beta}^{\bar{M}} - \boldsymbol{\beta}^M) - \mathbf{E}_{(N_{mis}^G,-k)}\boldsymbol{\beta}_{-k}^{\bar{M}}$ and $B = \mathbf{M}_{N_{mis}^G \times J}\Omega_k$. The complete gene expression data for gene k is

$$\boldsymbol{E}_k = (\boldsymbol{E}_{N_{obs}^G,k}^T, \boldsymbol{E}_{N_{mis}^G,k}^T)^T$$

- Samples with missing DNA methylation data are imputed as

$$\boldsymbol{M}_{N^M_{mis},j} \sim N(\sigma^2_M \omega_{jk}(A(\beta^{\bar{M}}_k - \beta^M_k)\sigma^{-2} + B\sigma^{-2}_k), \sigma^2_M)$$

for methylation probe $j \in \mathcal{J}_k$ where $\sigma^2_M = (1+\omega^2_{jk}(\sigma^{-2}_k+(\beta^{\bar{M}}_k-\beta^M_k)^2\sigma^{-2}))^{-1}$; $A = \boldsymbol{Y}_{N^M_{mis}} - \mathbf{C}_{N^M_{mis}}\boldsymbol{\beta}^C - \mathbf{M}_{(N^M_{mis},-j)}\Omega_{(-j,\cdot)}(\boldsymbol{\beta}^{\bar{M}} - \boldsymbol{\beta}^M) - \mathbf{E}_{N^M_{mis}}\boldsymbol{\beta}^{\bar{M}}$ and $B = \boldsymbol{E}_{N^M_{mis},k} - \mathbf{M}_{(N^M_{mis},-j)}\Omega_{-j,k}$. Then the complete DNA methylation for probe $j \in \mathcal{J}_k$ is

$$\boldsymbol{M}_j = (\boldsymbol{M}^T_{N^M_{obs},j}, \boldsymbol{M}^T_{N^M_{mis},j})^T$$

After the complete data is attained, the parameter estimation is conducted via the procedure in the section 2.1 and 2.2.

1.4 Updating latent variable for binary response and survival response

For binary response and survival response, we augment a latent continuous variable. In the MCMC procedure, we are required to update the latent variable.

- For binary response, we update the latent response variable Y^*_n from a truncated normal distribution.

$$Y^*_n \sim N(\mu_n, 1)\mathcal{I}(Y^*_n > 0) \quad \text{if} \quad Y_n = 1$$

$$Y^*_n \sim N(\mu_n, 1)\mathcal{I}(Y^*_n \leq 0) \quad \text{if} \quad Y_n = 0$$

where $\mu_n = \boldsymbol{c}^T_n\boldsymbol{\beta}^C + \boldsymbol{E}_n\boldsymbol{\beta}^{\bar{M}} + (\mathbf{M}\Omega)_n(\boldsymbol{\beta}^{\bar{M}} - \boldsymbol{\beta}^M)$.

- For survival response, we update the latent response Y^*_n corresponding to censored response ($\delta_n = 0$) by the Metropolis-Hastings algorithm. We implement the Metropolis-Hastings step with an exponential proposal distribution $q\left(Y^{*\,\text{new}}_n, Y^{*\,\text{old}}_n\right)$, with the scalar parameter λ and location parameter $\log(c_n)$. We set $\lambda = \left(y^{\star\,\text{old}}_n - \log(c_n)\right)^{-1}$ so that the expected value of the proposal distribution is $Y^{*\,\text{old}}_n$. We accept a new value with probability

$$\min\left\{\frac{P(Y^{*\,\text{new}}_n, \boldsymbol{Y}^*_{-n} \mid \Theta, \mathbf{C}, \mathbf{E}, \mathbf{M})q(Y^{*\,\text{old}}_n, Y^{*\,\text{new}}_n)}{P(Y^{*\,\text{old}}_n, \boldsymbol{Y}^*_{-n} \mid \Theta, \mathbf{C}, \mathbf{E}, \mathbf{M})q(Y^{*\,\text{new}}_n, Y^{*\,\text{old}}_n)}, 1\right\}$$

where Θ are the model parameters.

2 The uncertainty in the data augmentation

It is noticed that missing data imputation procedures may often decrease the model performance. Although we have designed a cross-validation framework to determine whether or not to contain missing data in the model, we also explored the reason behind the counter-intuitive phenomenon. Actually, the phenomenon widely exists in the missing data problem. We consider a simple problem of correlation estimation for bivariate normal data as an example.

In the problem, the observations (x, y) come from $N(\boldsymbol{\mu}, \boldsymbol{\Sigma})$ with parameter $\boldsymbol{\mu} = (\mu_x, \mu_y)^T$ and

$$\boldsymbol{\Sigma} = \begin{pmatrix} \sigma_x^2 & \rho\sigma_x\sigma_y \\ \rho\sigma_x\sigma_y & \sigma_y^2 \end{pmatrix}$$

However, in some, but not all, of the data pairs, either the x or the y is missing. Assume that n_{xy} data pairs are observed completely; n_x data pairs are missing in y and n_y data pairs are missing in x. The two estimators considered are $\hat{\rho}$ the maximum likelihood estimator using only complete data pairs and $\tilde{\rho}$ the maximum likelihood estimator using all the observations. The data is assumed to be *missing completely at random* (MCAR). Based on complete data pairs, it is well known that the MLE of ρ is

$$\hat{\rho} = \frac{\sum_{i=1}^{n_{xy}} \left(x_i - \bar{x}_{n_{xy}}\right)\left(y_i - \bar{y}_{n_{xy}}\right)}{\sqrt{\sum_{j=1}^{n_{xy}} (x_j - \bar{x}_{n_{xy}})^2 \sum_{k=1}^{n_{xy}} (y_k - \bar{y}_{n_{xy}})^2}}$$

For the asymptotic property of $\hat{\rho}$, it is well known that $\sqrt{n_{xy}}[\hat{\rho} - \rho] \xrightarrow{d} \mathrm{N}\left(0, \left[1 - \rho^2\right]^2\right)$ as $n_{xy} \to \infty$.

Based on all the observations, the data likelihood is

$$\begin{aligned} L(\boldsymbol{\theta}) =& (2\pi)^{-n_{xy}} |\boldsymbol{\Sigma}|^{-n_{xy}/2} \exp\left\{-\frac{1}{2}\sum_{i=1}^{n_{xy}} \left[(x_i, y_i)^T - \boldsymbol{\mu}\right]^T \boldsymbol{\Sigma}^{-1} \left[(x_i, y_i)^T - \boldsymbol{\mu}\right]\right\} \\ &\times \left(2\pi\sigma_x^2\right)^{-n_x/2} \exp\left\{-\frac{1}{2}\sum_{j=1}^{n_x} \left[\left(x_j^* - \mu_x\right)/\sigma_x\right]^2\right\} \\ &\times \left(2\pi\sigma_y^2\right)^{-n_y/2} \exp\left\{-\frac{1}{2}\sum_{k=1}^{n_y} \left[\left(y_k^* - \mu_y\right)/\sigma_y\right]^2\right\}, \end{aligned}$$

There is no analytic solution for the estimator $\tilde{\rho}$. We can implement some numerical optimization algorithms such as the gradient descent algorithm to obtain the numerical solution. According to the results of Garren (1998), it is shown that the numerical solution $\tilde{\rho}$ have

$$\sqrt{n_{xy}}[\tilde{\rho}-\rho] \xrightarrow{d} \mathrm{N}\left(0, \left[1+\rho^2\right]^{-1}\left[1-\rho^2\right]^2\right)$$

as $n_{xy} \longrightarrow \infty$, where $n_{xy} = o(\min\{n_x, n_y\})$. Since the normal distribution has a smaller variance compared to estimators using complete data pairs, it seems that the estimator will be more accurate when including all the observations. However, in their simulation, they discovered that when the condition $n_{xy} = o(\min\{n_x, n_y\})$ was violated greatly, the accuracy of estimators using all the observations would decrease. The efficiency of $\tilde{\rho}$ seemed to improve as n_x and n_y got large. The efficiency of $\hat{\rho}$ tended to increase as n_{xy} got large.

The rule is also consistent with the discovery in our model. As the complete data size becomes large, the model only using the complete data will perform better. However, we cannot determine the threshold of sample size for complete data. The cross-validation scheme will help us determine whether or not to include the missing data in the model.

3 Additional simulation results

3.1 Sensitivity analysis

Since the clinical covariates C are always included in the model, we set $\tau_c = 0.001$, giving a flat prior. The hyperparameter (a, b) in the prior of $\pi_{1:K}$ is $(a, b) = (0.2, 0.8)$, indicating average 20% methylation probes are effective. For the hyperparameter (δ_1, δ_2) in the prior of model variance, vague prior is assigned with $(\delta_1, \delta_2) = (0.001, 0.001)$. To assess the influence of hyperparameters $\tau, \tau_{1:K}, d, f$ on the posterior inference, we applied our method on the same simulation data set generated from the simulation scheme of scenario 1.

We report here the sensitivity results on the choice of the MRF prior hyperparameters, d and f, and of the hyperparameters τ and $\tau_{1:K}$ in the spike-and-slab prior. Tables S1-S4 show averaged

sensitivity (Sens), specificity (Spec), Matthews correlation coefficients (MCC), and area under ROC curves (AUC) based on the PPI estimates of $\boldsymbol{\gamma}$, averaged over 50 simulated datasets. The definitions of sensitivity, specificity and MCC are

$$\text{Sensitivity} = \frac{\text{TP}}{\text{TP} + \text{FN}}$$
$$\text{Specificity} = \frac{\text{TN}}{\text{TN} + \text{FP}}$$
$$\text{MCC} = \frac{\text{TP} \times \text{TN} - \text{FP} \times \text{FN}}{\sqrt{(\text{TP} + \text{FP})(\text{TP} + \text{FN})(\text{TN} + \text{FP})(\text{TN} + \text{FN})}}$$

According to the results in Table S1-S4, when fixing f, the results are robust for small values of d, although the sensitivity decreases slightly. On the contrary, when fixing d, the results are sensitive to the choice of f. Smaller values of f decrease the ability to identify true significant features, but larger values of f select many nonsignificant features as a result of the phase transition phenomenon in the MRF prior. Hence, moderate values of f, 0.5, and 1, are preferred in the analysis. Finally, the choice of d and f does not influence the selection of DNA methylation probes since the effects from methylation to gene expression are not associated with the effects from gene expression to clinical response. As for the choice of hyperparameter τ, larger values of τ increase the penalty on the regression coefficients, resulting in lower sensitivity and higher specificity. Generally, when $\tau \in [1, 10]$, the feature selection results are robust. The feature selection results are robust to the choice of hyperparameters $\tau_{1:K}$.

3.2 Comparison with imputation method

We compared the NetMIM with a two-stage method based on missing data imputation, defined as NetMIM_IM. In the first stage, we imputed the missing entries of multi-omics data with R package `mice`. The number of multiple imputations was 5. In the second stage, we implemented NetMIM on the imputed data set. The performance of NetMIM, NetMIM_CC, and NetMIM_IM are shown in Figure S1, which illustrates that NetMIN_CC always performed better on feature selection and model prediction than NetMIN_IM when gene expression was missing. When DNA methylation was missing, NetMIM performed better than NetMIM_IM under high missing ratios.

3.3 Evaluation for random graph

In order to verify that the genes selected in NetMIM are not selected only thanks to a regularization effect but that the graph also plays an important part in this process, we randomly permuted the edges in the original graph. The new method is defined as NetMIM w/ Rand_Net. The NetMIM w/ Rand_Net was implemented on the data generated in the simulation scenario I. The feature selection and model prediction performance of NetMIM, NetMIM w/o MRF, and NetMIM w/ Rand_Net are shown in Figure S2. In most cases, NetMIM w/ Rand_Net performed worse than NetMIM w/ MRF under feature selection and model prediction. It implies that a wrong information prior is worse than a noninformative prior. Thus, the graph structure plays an important role in the feature selection procedure instead of via the effect of regularization.

4 Additional results for KIRC study

In our model formulation, the specification of MRF requires a network among different genes. A gene-gene interaction network was extracted from the KEGG database. We included 29 pathways identified by Yang et al. (2014) and Yuan et al. (2018), where differential genes were enriched in the Kidney Renal Clear Cell Carcinoma (KIRC). The pathways are listed below: Taurine and hypo-taurine metabolism; Neuroactive ligand-receptor interaction; Glycosaminoglycan biosynthesis - heparin-sulfate; Peroxisome proliferator-activated receptor (PPAR) signaling pathway; Hepatitis C; Gastric acid secretion; Primary immunodeficiency; Small cell lung cancer; Bladder cancer; Pancreatic cancer; hsa05200; Aldosterone-regulated sodium reabsorption; Leukocyte transendothelial migration; Hematopoietic cell lineage; Complement and coagulation cascades; Cell adhesion molecules (CAMs); Focal adhesion; Axon guidance; p53 signaling pathway; Cytokine-cytokine receptor interaction; ABC transporters; Butanoate metabolism; Arachidonic acid metabolism; Arginine and proline metabolism; Valine, leucine and isoleucine degradation; Glycine, serine and threonine metabolism; Fructose and mannose metabolism; Citrate cycle (TCA cycle); Glycolysis.

There was a total of 1772 genes in these pathways. TPM of RNA sequence was used as the gene expression level. After filtering out genes with mean expression levels less than 10 or standard deviation less than 5, we included 814 genes in the model. The network among the

814 genes was extracted using the R package `KEGGgraph` of Zhang and Wiemann (2009).

4.1 Cross-validation results

We randomly split the complete training dataset into 5 folds, keeping the censoring ratio almost the same in each fold. Each fold was selected as the validation dataset with the remaining samples used as the training data. The method NetMIM_CC was trained on the complete omics data and NetMIM was trained on the omics data of all subjects. Table S5 showed that incorporating samples without DNA methylation improved the model performance. Hence, we always included the samples without DNA methylation in the downstream analysis.

4.2 Algorithm configuration of other methods

IPF_Lasso was performed via the R package `ipflasso` (Boulesteix et al., 2017). Every type of omics data was input as a block, and the penalty factors for different blocks were selected through 5-fold cross validation. Block forest was fitted via R package `blockForest` (Hornung and Wright, 2019). Block information of different omics data was provided. The parameters were set as nsets = 300, num.trees.pre =1500 and num.trees = 2000 as suggested. DeepOmix (Zhao et al., 2021) was implemented with default settings in their tutorials with learning rate = 0.003 and penalty parameter determined via 5-fold cross-validation.

4.3 MCMC diagnostic of case study

We implemented the Bayesian algorithm on the training dataset incorporating samples without DNA methylation. We conducted four different chains from different initials. The Gelman and Rubin statistics for the posterior of the four MCMC chains was 1.04 as shown in Figure S6. The values of Gelman and Rubin statistics for intercept, coefficient of age, coefficient of gender, and the variance in the clinical model are 1.01, 1.01, 1.02, and 1.06, respectively (Figure S7). It implied that our algorithm converged well. We also compared the gene selection results and probe selection results between the proposed method with MRF prior and without MRF prior. Without MRF prior, only two genes were identified to be associated with the patient's survival.

4.4 Biological findings

In order to validate the biological relevance of our discoveries, we focused on the gene list identified by our model associated with the patient's survival time. Firstly, we conducted enrichment analysis for the total 175 identified genes by employing databases for annotation visualization and integrated discovery (DAVID) (Dennis et al., 2003). The annotation terms at the 0.05 threshold of adjusted p-values were selected. Secondly, we performed a literature survey on the 14 genes possessing both type M effect and type $\bar{M}$ effect, which is more likely to play a role in cancer regulations.

We have identified several significant annotation terms. For the pathway category, under the KEGG pathway sub-category (Figure S11), the pathway Focal adhesion shows a relatively big gene ratio and the smallest adjusted p-value (1.5×10^{-140}). In contrast to previous enrichment analyses of differentially expressed genes (DEGs) in the literature, where Focal adhesion did not rank among the top ten significantly enriched pathways, our analysis reveals that Focal adhesion emerges as a high-ranking pathway. Proteins in the Focal adhesion are associated with cancer metastasis, which is responsible for as many as 90% of cancer-associated deaths in patients (Gorka et al., 2022). There are a lot of reports about the regulation of metastasis in renal clear cell carcinoma. The terms PI3K-Akt signaling pathway and pathways in cancer followed by the second and third smallest adjusted p-values (7.8×10^{-77} and 2.8×10^{-73}, respectively). The PI3K-Akt signaling pathway is an intracellular signaling pathway, which is important in regulating the cell cycle and central regulator pathway of several cancers, such as ovarian cancer and breast cancer. Secondly, we found significant terms in the gene ontology (GO) category for biological process (BP), cellular component (CC), and molecular function (MF). For the biological process in the GO terms, positive regulation of cell migration, cell adhesion, positive regulation of cell proliferation, cell migration, integrin-mediated signaling pathway, and cell-matrix adhesion were enriched terms, most of which were associated with cell migration, resulting in the metastasis of cancer cells. Among the identified cellular components, focal adhesion and integrin complex were the most significant terms. Integrins are transmembrane receptors that facilitate the cell-cell and cell-extracellular matrix (ECM) adhesion, regulating the cell cycle upon the ligand binding. In the last term, molecular function, integrin bind showed the smallest adjusted p-value (8.4×10^{-27}). It also implied the function

of integrins played an important role in the development of Kidney Renal Clear Cell Carcinoma.

There were 14 identified genes with both type M effect and type $\bar{M}$ effect in our model, the symbols of which are CLDN3, CLDN5, EGFR, HRAS, KRAS, AKT2, CCND1, MYC, ESAM, COL6A3, THBS3, VMF, IGF1R, MET. We performed a literature search for the 14 genes, confirming the relevance of our results. For example, the claudin (CLDN) genes encode a family of proteins that are important in tight junction formation, which are associated with metastasis of cancer cells (Hewitt et al., 2006). The Ras gene family is a famous oncogene, related to the initialization and progression of cancers. The cyclin family (CCND1) forms a complex and functions as a regulatory subunit of CDK4 or CDK6, whose activity is required for cell cycle G1/S transition. MYC is a family of regulator genes or transcription factors, involved in cell proliferation, contributing to the formation of cancer. For THBS3, recent studies have shown that the THBS family plays a vital role in the development and progression of human cancer. Especially, the over-expression of the THBS family protein will decrease the survival time of cancer patients, which is consistent with our discoveries. The growth factors and their receptors (EGFR, IGF1R, MET) correlate with poor prognosis and the formation of new blood vessels that supply the tumor with nutrients.

5 Analysis of LUAD study

We also apply the proposed method to lung adenocarcinoma (LUAD) data from The Cancer Genome Atlas (TCGA) data portal, with DNA methylation data from Illumina 450K chips and RNA-seq gene expression data. The dataset includes $N = 487$ lung cancer patients, 3 of whom have no gene expression data, and 63 of whom have no DNA methylation data, implying that almost 14% of the subjects do not have full omics data. For genetic features, we are interested in the genes belonging to 35 KEGG pathways in the enrichment analysis of differential genes for LUAD, resulting in 1995 genes Zengin and Önal-Süzek (2021). The pathways are listed below: Chemokine signaling pathway; PI3K-Akt signaling pathway; Human papillomavirus infection; Rap1 signaling pathway; Ras signaling pathway; Focal adhesion; Platelet activation; ECM-receptor interaction; Phospholipase D signaling pathway; Cushing syndrome; Circadian entrainment; Gastric cancer; AGE-RAGE signaling pathway in diabetic compli-

cations; Melanogenesis; Relaxin signaling pathway; Cytokine-cytokine receptor interaction; Insulin secretion; Signaling pathways regulating pluripotency of stem cells; Progesterone-mediated oocyte maturation; Proteoglycans in cancer; Cell cycle; Kaposi sarcoma-associated herpesvirus infection; Oocyte meiosis; Small cell lung cancer; Salivary secretion; Vascular smooth muscle contraction; Apelin signaling pathway; Aldosterone synthesis and secretion; Pancreatic secretion; Amoebiasis; Hepatocellular carcinoma; Human T-cell leukemia virus 1 infection; Gap junction; Adrenergic signaling in cardiomyocytes; JAK-STAT signaling pathway.

The RNA-seq counts are transformed into continuous TPM (transcripts per million) values, and DNA methylation levels are represented by the M-value. We filter out genes with a mean expression level less than 10 or a standard deviation less than 5, resulting in $K = 1010$ genes for the analysis. In addition, we select the methylation probes mapped to each gene in the promoter region, obtaining $J = 16379$ methylation probes. In our model formulation, the last component, MRF prior on selection indicators γ, requires the interaction network $\mathbf{G}$ among $K = 1010$ genes, which are extracted from the KEGG database with the `R` package `KEGGgraph` (Zhang and Wiemann, 2009). If there exists a direct interaction in a pathway for gene j and gene l, then $g_{jl} = 1$; $g_{jl} = 0$ otherwise. To compare the performance of the proposed method including subjects with missing omics data and complete data, we randomly split the complete data into training data (338) and test data (83) after removing subjects with a survival time less than 30 days. The censoring proportions in each subset are the same (64%). For prior specification, the hyperparameters to control the MRF prior are set as $d = -4$ and $f = 0.5$ to assign a prior probability of inclusion of genes as approximately 0.02. The other hyperparameters are the same as the simulation studies.

5.1 Results

We computed the pairwise Pearson correlation coefficients of the marginal posterior probability of inclusion for $\boldsymbol{\gamma}$ between different chains to check the consistency of selection results. The correlation coefficients of the posterior probability of inclusion ranged from 0.853 to 0.921 for $\boldsymbol{\gamma}$ and from 0.876 to 0.896 for $\mathbf{Z}$ among the four chains, demonstrating good convergence and consistent variable selection results of our model. Furthermore, we compared the performance

of NetMIM, NetMIM_CC, NetMIM_CV, and these methods without MRF prior. DeepOmix, BlockForest, and IPF_LASSO were implemented on the complete dataset. As shown in Table S6, NetMIM_CC performed better on both the test datasets. Regarding the efficiency, the computational time was 10.3 hours, 3.0 hours, 6.4 mins, and 2.9 mins for NetMIM_CC, DeepOmix, BlockForest, and IPF_LASSO respectively.

Applying the NetMIM_CC model to the training data, we identified 108 genes with only *type M effect*, 43 genes with only *type* $\bar{M}$ *effect*, and 8 genes with both *type M effect* and *type* $\bar{M}$ *effect* when utilizing the median probability model for selection (PPI cutoff = 0.5). This result implies that the 8 genes have effects modulated by both methylation and other mechanisms. Additionally, we identified 187 genes that are significantly modulated by at least one methylation probe mapped to their promoter regions with a threshold equal to 0.5.

5.2 Biological findings

To gain further insights into the identified genes, we performed an enrichment analysis using the databases for annotation visualization and integrated discovery (DAVID) (Dennis et al., 2003). We selected annotation terms with adjusted p-values below the threshold of 0.05. Within the KEGG pathway sub-category, we observed that the top three enriched pathways were the PI3K-Akt signaling pathway (p-value = 1.3×10^{-45}), Pathways in cancer (p-value = 5.9×10^{-44}), and Focal adhesion (p-value = 1.0×10^{-43}), as depicted in Figure S12. Interestingly, these three pathways were also identified as the top three enriched pathways in the KIRC study. This finding further underscores the importance of the PI3K-Akt signaling pathway and Focal adhesion in the development and progression of human cancers. The consistency of these enriched pathways across different studies highlights their potential as key players in cancer-related processes.

References

Boulesteix, A.-L., De Bin, R., Jiang, X., Fuchs, M. et al. (2017), IPF-LASSO: integrative-penalized regression with penalty factors for prediction based on multi-omics data, *Computational and Mathematical Methods in Medicine* **2017**.

Dennis, G., Sherman, B. T., Hosack, D. A., Yang, J., Gao, W., Lane, H. C. and Lempicki, R. A. (2003), David: database for annotation, visualization, and integrated discovery, *Genome Biology* **4**(9), 1–11.

Garren, S. T. (1998), Maximum likelihood estimation of the correlation coefficient in a bivariate normal model with missing data, *Statistics & Probability Letters* **38**(3), 281–288.

Gorka, J., Marona, P., Kwapisz, O., Rys, J., Jura, J. and Miekus, K. (2022), Mcpip1 regulates focal adhesion kinase and rho gtpase-dependent migration in clear cell renal cell carcinoma, *European Journal of Pharmacology* **922**, 174804.

Hewitt, K. J., Agarwal, R. and Morin, P. J. (2006), The claudin gene family: expression in normal and neoplastic tissues, *BMC Cancer* **6**(1), 1–8.

Hornung, R. and Wright, M. N. (2019), Block forests: random forests for blocks of clinical and omics covariate data, *BMC Bioinformatics* **20**, 1–17.

Yang, W., Yoshigoe, K., Qin, X., Liu, J. S., Yang, J. Y., Niemierko, A., Deng, Y., Liu, Y., Dunker, A. K., Chen, Z. et al. (2014), Identification of genes and pathways involved in kidney renal clear cell carcinoma, *BMC Bioinformatics* **15**(17), 1–10.

Yuan, L., Zeng, G., Chen, L., Wang, G., Wang, X., Cao, X., Lu, M., Liu, X., Qian, G., Xiao, Y. et al. (2018), Identification of key genes and pathways in human clear cell renal cell carcinoma (ccrcc) by co-expression analysis, *International Journal of Biological Sciences* **14**(3), 266.

Zengin, T. and Önal-Süzek, T. (2021), Comprehensive profiling of genomic and transcriptomic differences between risk groups of lung adenocarcinoma and lung squamous cell carcinoma, *Journal of Personalized Medicine* **11**(2), 154.

Zhang, J. D. and Wiemann, S. (2009), Kegggraph: a graph approach to kegg pathway in r and bioconductor, *Bioinformatics* **25**(11), 1470–1471.

Zhao, L., Dong, Q., Luo, C., Wu, Y., Bu, D., Qi, X., Luo, Y. and Zhao, Y. (2021), Deepomix: A scalable and interpretable multi-omics deep learning framework and application in cancer survival analysis, *Computational and Structural Biotechnology Journal* **19**, 2719–2725.

Table S1: Sensitivity analysis on the choice of hyperparameters d and f in the MRF prior: Means and standard deviations (in parentheses) of sensitivity, specificity, MCC and area under the ROC curves (AUCs) on the posterior probability of inclusion for $p(\gamma_k^M = 1 \mid \cdot)$ and $p(\gamma_k^{\bar{M}} = 1 \mid \cdot)$. A threshold of 0.5 was utilized. Results are averages over 50 simulated datasets.

d	f	Sens	Spec	MCC	AUC
-1	1	0.843 (0.038)	0.833 (0.029)	0.371 (0.042)	0.927 (0.026)
-2	1	0.844 (0.039)	0.969 (0.011)	0.691 (0.065)	0.942 (00.27)
-3	1	0.832 (0.046)	0.992 (0.006)	0.830 (0.052)	0.936 (0.026)
-4	1	0.824 (0.045)	0.996 (0.003)	0.864 (0.038)	0.924 (0.020)
-5	1	0.811 (0.052)	0.997 (0.003)	0.867 (0.042)	0.924 (0.035)
-3	0	0.778 (0.049)	0.995 (0.004)	0.826 (0.044)	0.938 (0.027)
-3	0.5	0.805 (0.054)	0.995 (0.004)	0.846 (0.054)	0.949 (0.028)
-3	1.5	0.826 (0.046)	0.986 (0.007)	0.786 (0.056)	0.916 (0.028)
-3	2	0.818 (0.048)	0.970 (0.010)	0.682 (0.068)	0.904 (0.032)

Table S2: Sensitivity analysis on the choice of hyperparameters d and f in the MRF prior: Means and standard deviations (in parentheses) of sensitivity, specificity, MCC and area under the ROC curves (AUCs) on the posterior probability of inclusion for $p(Z_j = 1 \mid \cdot)$. A threshold of 0.5 was utilized. Results are averages over 50 simulated datasets.

d	f	Sens	Spec	MCC	AUC
-3	0	0.921 (0.015)	0.930 (0.009)	0.849 (0.018)	0.958 (0.006)
-3	0.5	0.921 (0.014)	0.930 (0.009)	0.849 (0.018)	0.959 (0.006)
-3	1	0.920 (0.014)	0.930 (0.008)	0.848 (0.017)	0.958 (0.006)
-3	1.5	0.920 (0.015)	0.931 (0.009)	0.849 (0.018)	0.958 (0.006)
-3	2	0.921 (0.013)	0.930 (0.009)	0.849 (0.016)	0.958 (0.006)
-2	1	0.920 (0.015)	0.931 (0.009)	0.849 (0.017)	0.958 (0.006)
-4	1	0.921 (0.014)	0.930 (0.009)	0.849 (0.017)	0.959 (0.007)

Table S3: Sensitivity analysis on the choice of hyperparameters τ in the prior of slab distribution of clinical model: Means and standard deviations (in parentheses) of sensitivity, specificity, MCC and area under the ROC curves (AUCs) on the posterior probability of inclusion for $p(\gamma_k^M = 1 \mid \cdot)$ and $p(\gamma_k^{\bar{M}} = 1 \mid \cdot)$. A threshold of 0.5 was utilized. Results are averages over 50 simulated datasets.

τ	**Sens**	**Spec**	**MCC**	**AUC**
0.1	0.834 (0.050)	0.986 (0.007)	0.791 (0.062)	0.938 (0.036)
1	0.832 (0.045)	0.992 (0.005)	0.831 (0.052)	0.936 (0.025)
2	0.815 (0.052)	0.994 (0.004)	0.846 (0.053)	0.932 (0.035)
5	0.808 (0.055)	0.997 (0.002)	0.869 (0.044)	0.917 (0.034)
10	0.797 (0.047)	0.998 (0.002)	0.869 (0.035)	0.908 (0.036)

Table S4: Sensitivity analysis on the choice of hyperparameters $\tau_0 = \tau_1 = \cdots = \tau_K$ in the prior of slab distribution of mechanistic model: Means and standard deviations (in parentheses) of sensitivity, specificity, MCC and area under the ROC curves (AUCs) on the posterior probability of inclusion for $p(Z_j = 1 \mid \cdot)$. A threshold of 0.5 was utilized. Results are averages over 50 simulated datasets.

τ_0	**Sens**	**Spec**	**MCC**	**AUC**
0.1	0.879 (0.017)	0.952 (0.007)	0.838 (0.018)	0.958 (0.007)
1	0.920 (0.014)	0.930 (0.008)	0.848 (0.017)	0.958 (0.006)
2	0.929 (0.015)	0.922 (0.009)	0.847 (0.019)	0.959 (0.006)
5	0.935 (0.011)	0.913 (0.011)	0.842 (0.019)	0.958 (0.006)
10	0.932 (0.013)	0.906 (0.012)	0.832 (0.020)	0.957 (0.006)

Table S5: The C-index of cross-validation results in the KIRC study.

Method	fold1	fold2	fold3	fold4	fold5	average
NetMIM	0.797	0.754	0.670	0.721	0.683	0.725
NetMIM_CC	0.630	0.727	0.676	0.700	0.662	0.679

Table S6: C-indices of training data and test data of different methods on LUAD data. DeepOmix, BlockForest, and IPF_LASSO are implemented on the complete data set.

Method	Training data	Test data
NetMIM_CC	**0.889 (0.033)**	**0.668 (0.033)**
NetMIM	0.815 (0.024)	0.655 (0.047)
NetMIM_CV	0.848 (0.037)	0.660 (0.064)
NetMIM_CC w/o MRF	0.772 (0.019)	0.659 (0.047)
NetMIM w/o MRF	0.767 (0.016)	0.647 (0.061)
NetMIM_CV w/o MRF	0.771 (0.015)	0.656 (0.069)
DeepOmix	0.843 (0.029)	0.663 (0.043)
BlockForest	0.516 (0.036)	0.509 (0.067)
IPF_LASSO	0.498 (0.027)	0.501 (0.053)

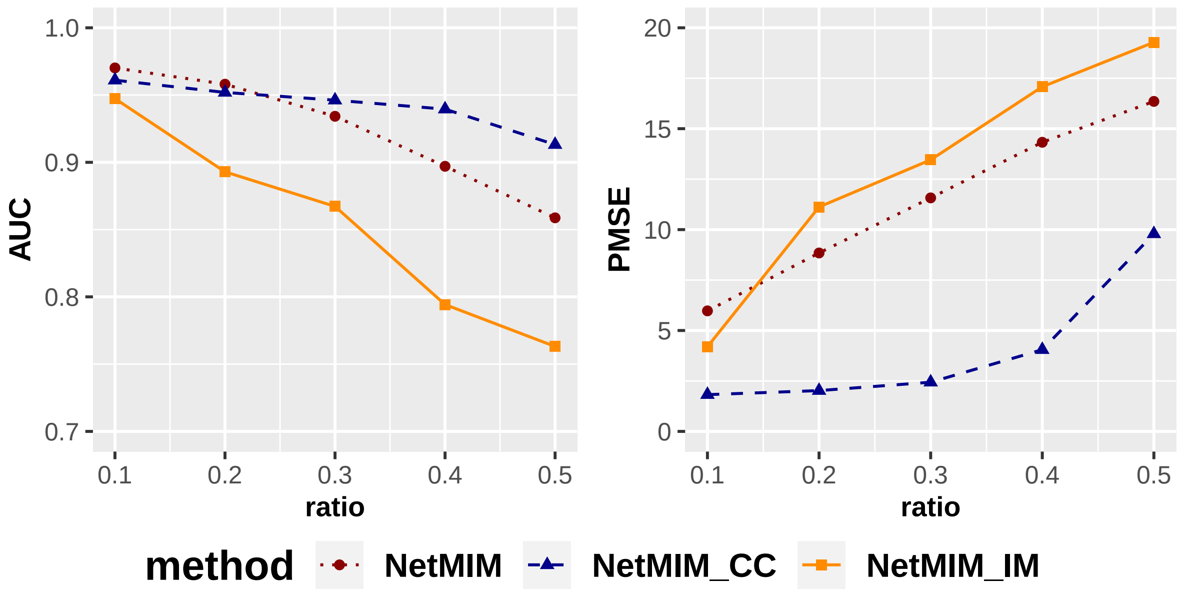

(a)

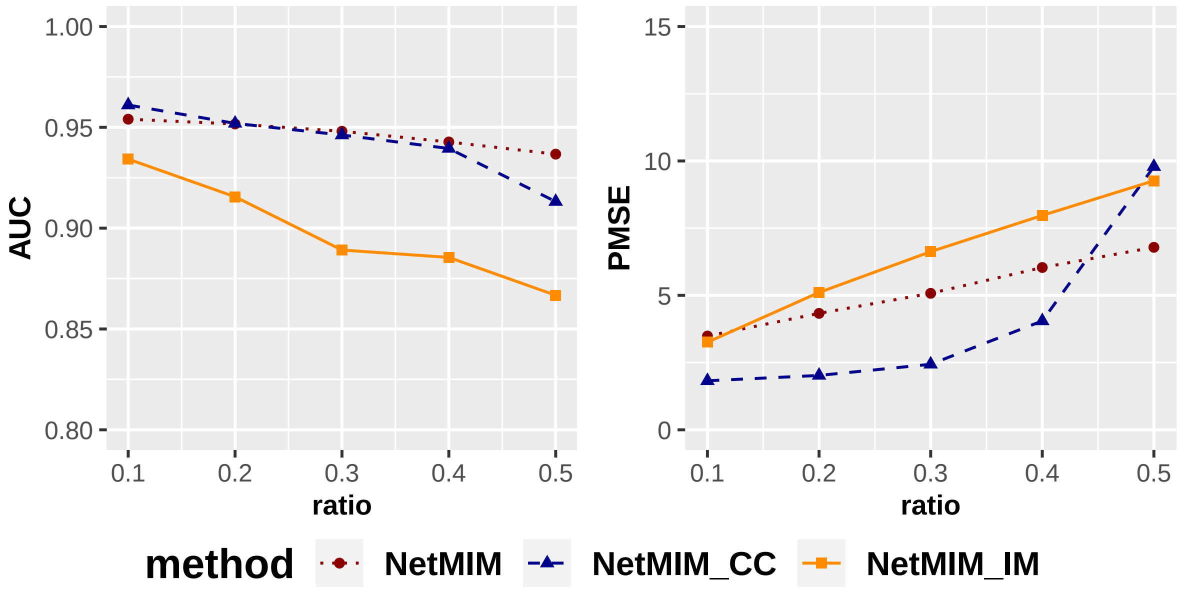

(b)

Figure S1: (a) AUC and PMSE of NetMIN, NetMIN_CC, and NetMIM_IM under $K = 100$ genes and different missing ratios for gene expression. (b) AUC and PMSE of NetMIN, NetMIN_CC, and NetMIM_IM under $K = 100$ genes and different missing ratios for DNA methylation.

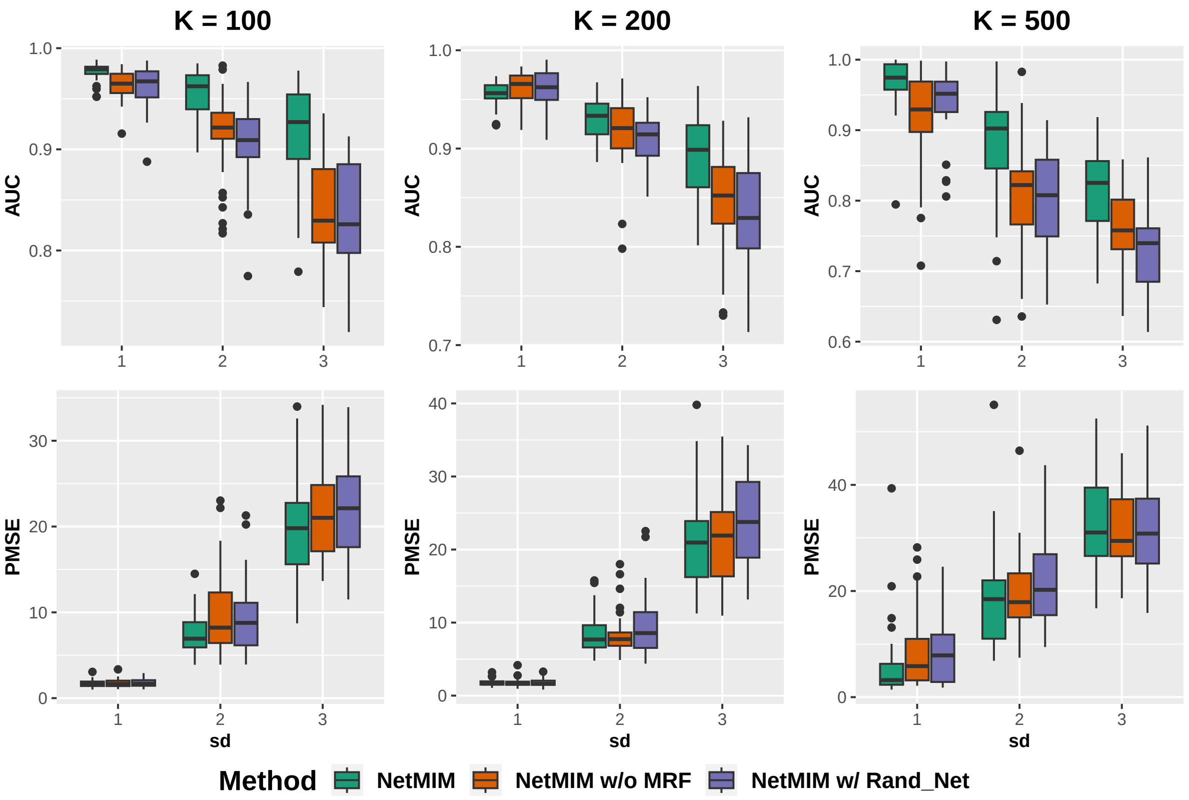

Figure S2: AUC and PMSE of NetMIM, NetMIM w/o MRF, and NetMIM w/ Rand_Net under different numbers of genes and model standard deviations, respectively. The number of genes K varies from 100, 200, 500. The x-axis represents the standard deviation σ of the clinical model.

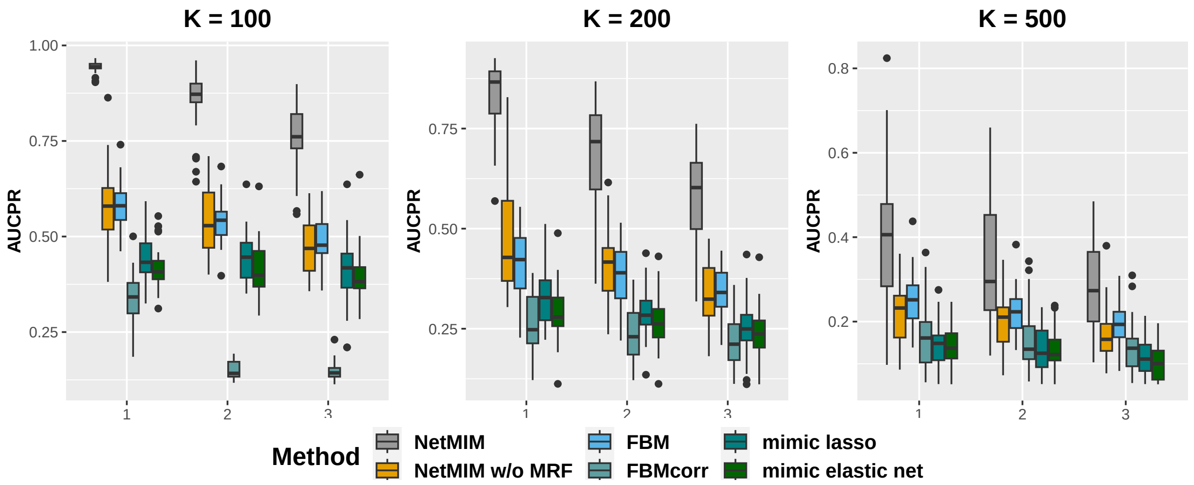
K = 100
K = 200
K = 500
AUCPR
Method
NetMIM
NetMIM w/o MRF
FBM
FBMcorr
mimic lasso
mimic elastic net

(a)

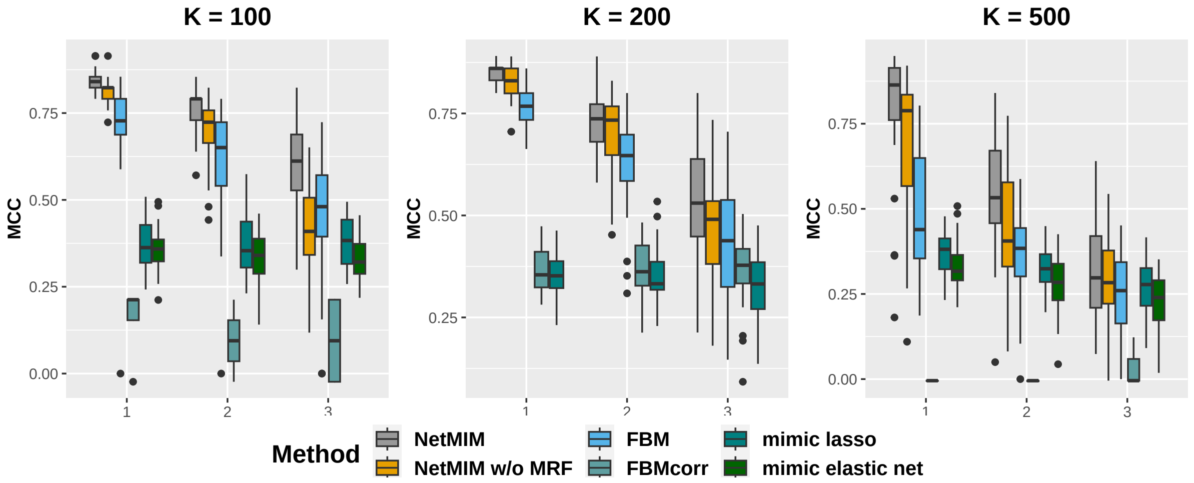
K = 100
K = 200
K = 500
MCC
Method
NetMIM
NetMIM w/o MRF
FBM
FBMcorr
mimic lasso
mimic elastic net

(b)

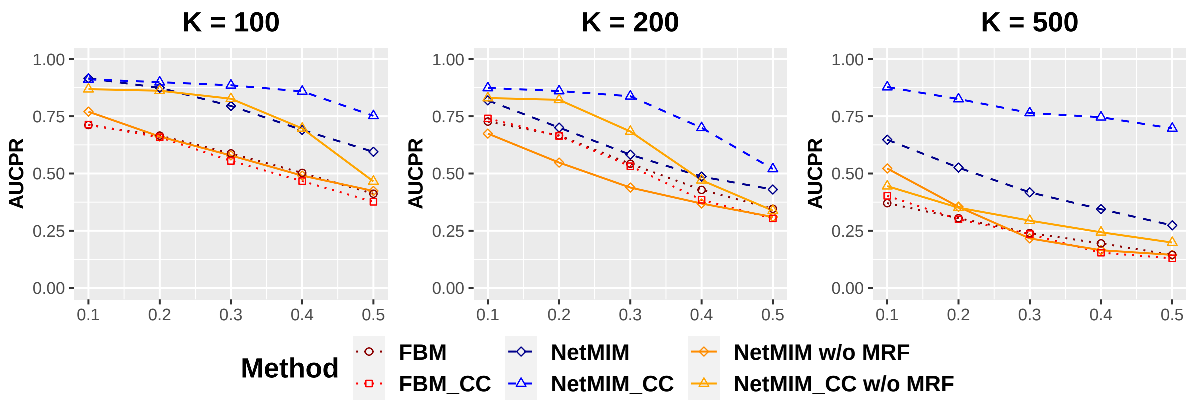

(c)

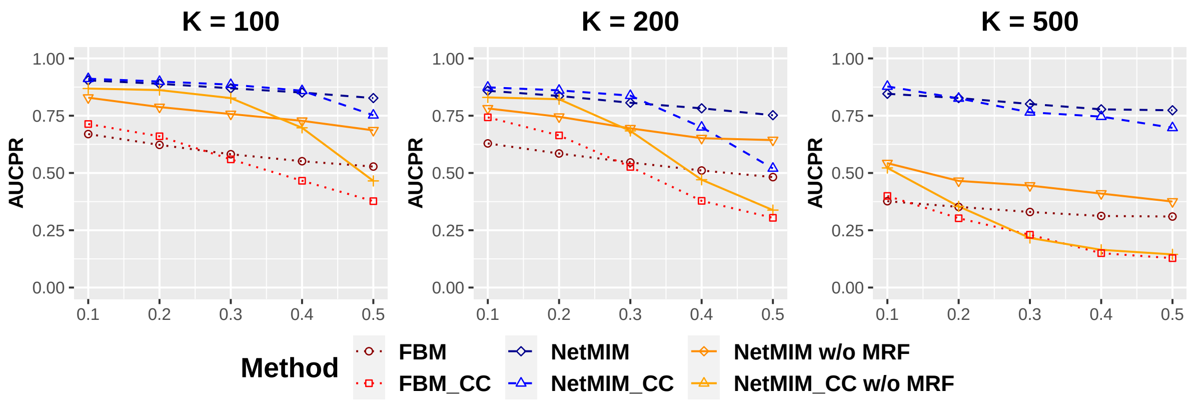

(d)

Figure S3: (a) AUCPR different methods under different numbers of genes and model standard deviations, respectively. (b) MCC different methods under different numbers of genes and model standard deviations, respectively. (c) AUCPR of different methods under different missing ratios for gene expression. (d) AUCPR of different methods under different missing ratios for DNA methylation expression.

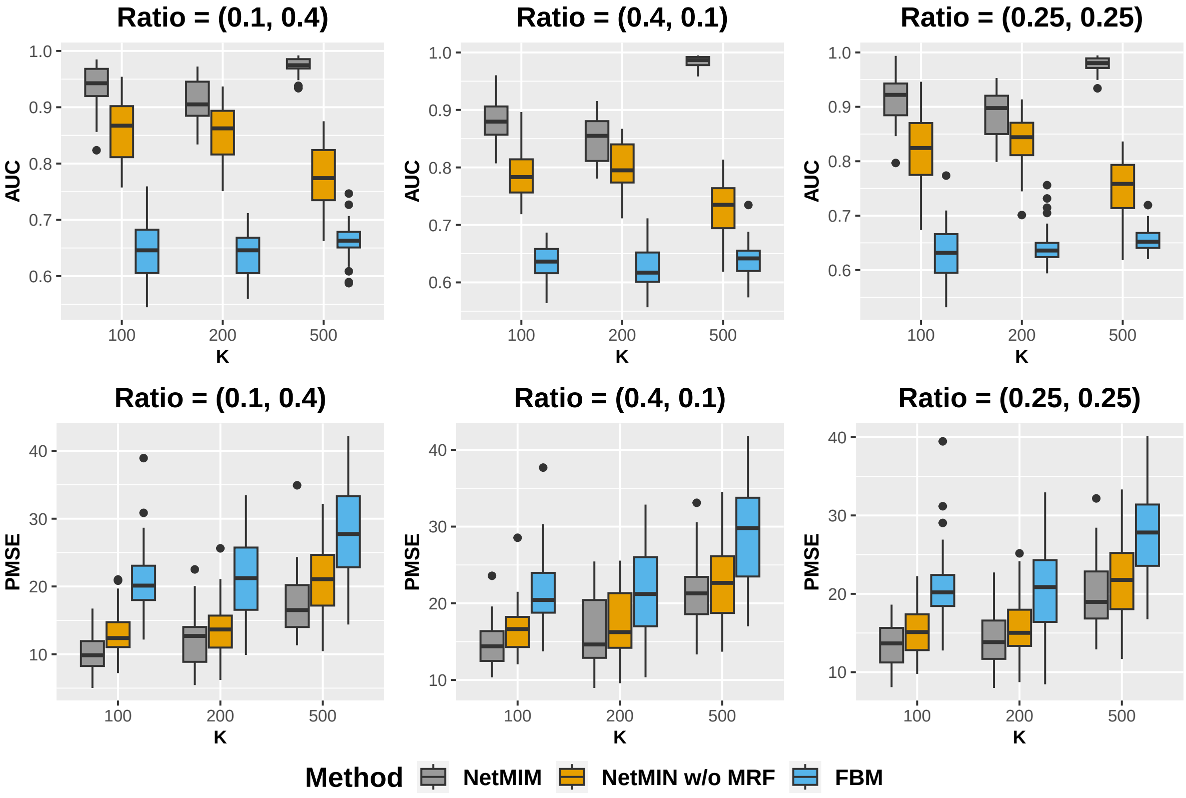

Figure S4: AUC and PMSE of different methods when both gene expression and DNA methylation are missed in some subjects. The first entry within the bracket is the proportion of subjects without gene expression and the second entry is that without DNA methylation. The number of genes K varies from 100, 200, 500.

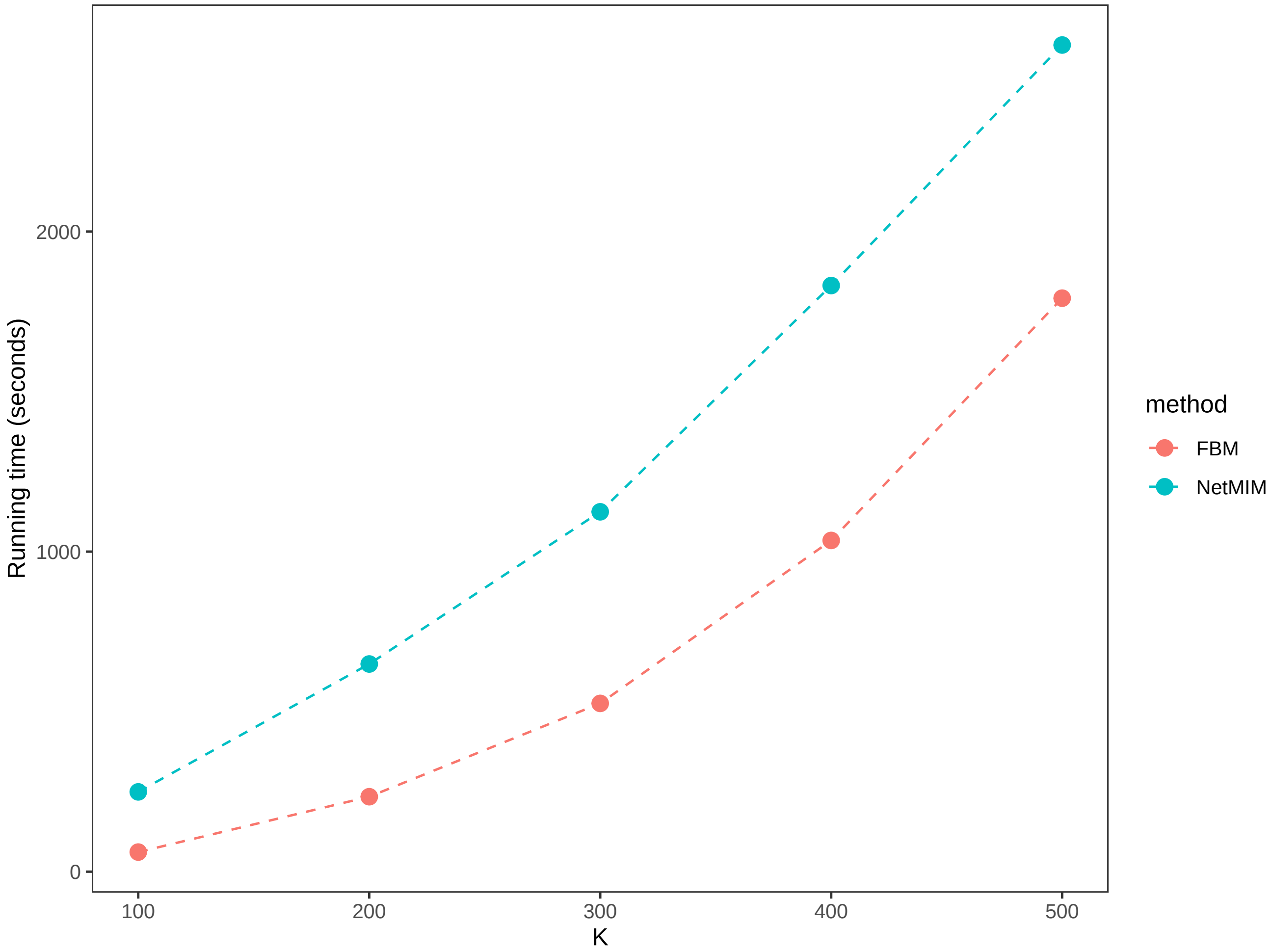

Figure S5: Computational time in seconds of NetMIM and FBM in the simulation scenario I, where K is the number of genes.

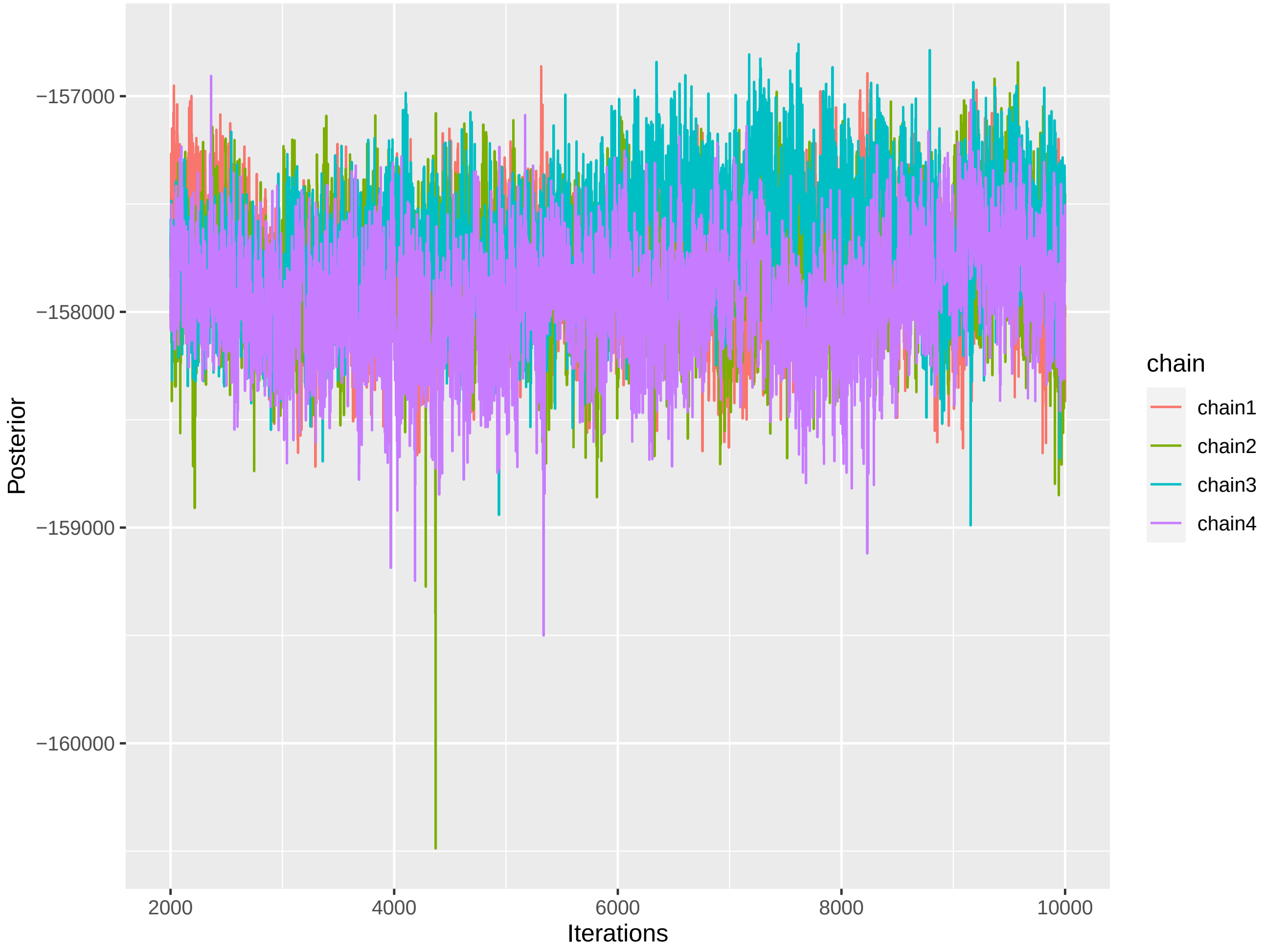

Figure S6: KIRC study: Trace plot of posterior for four different chains.

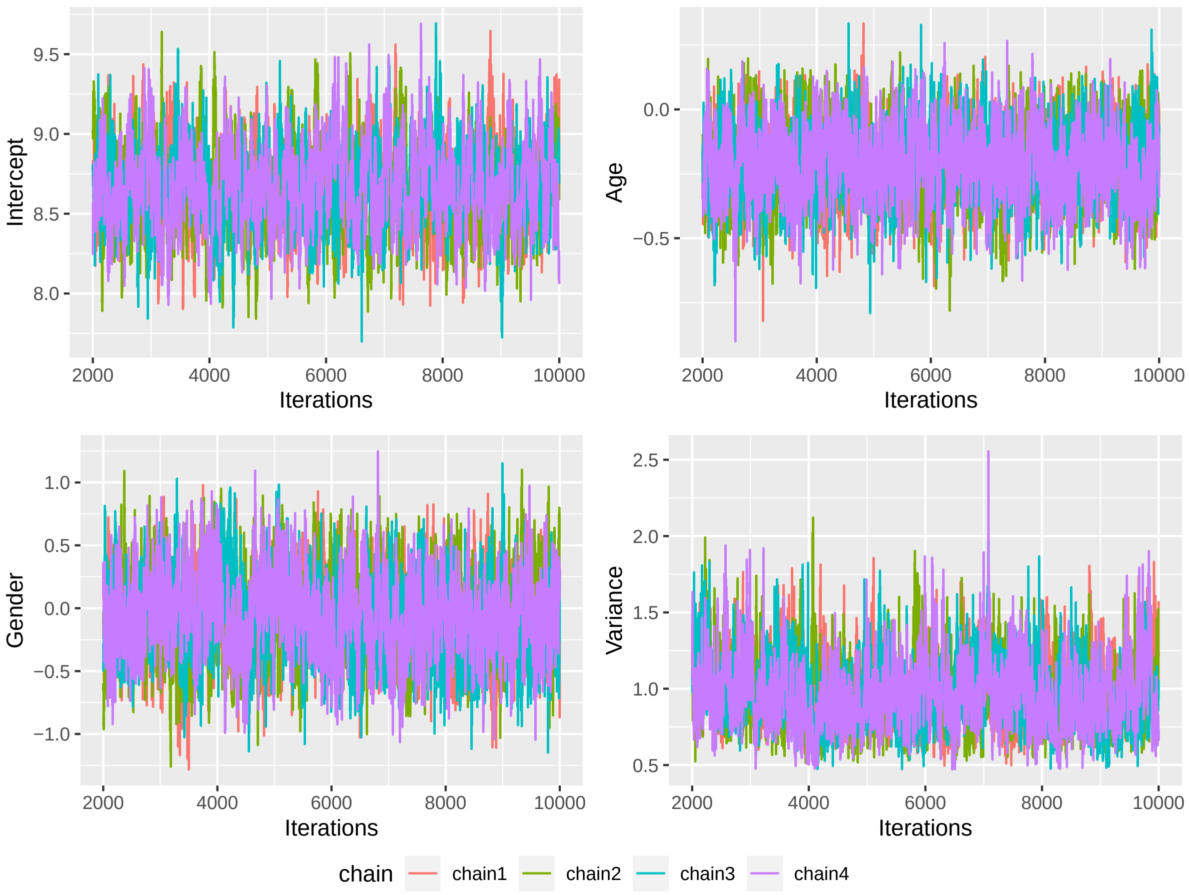

Figure S7: KIRC study: Trace plot of parameters in the four different chains.

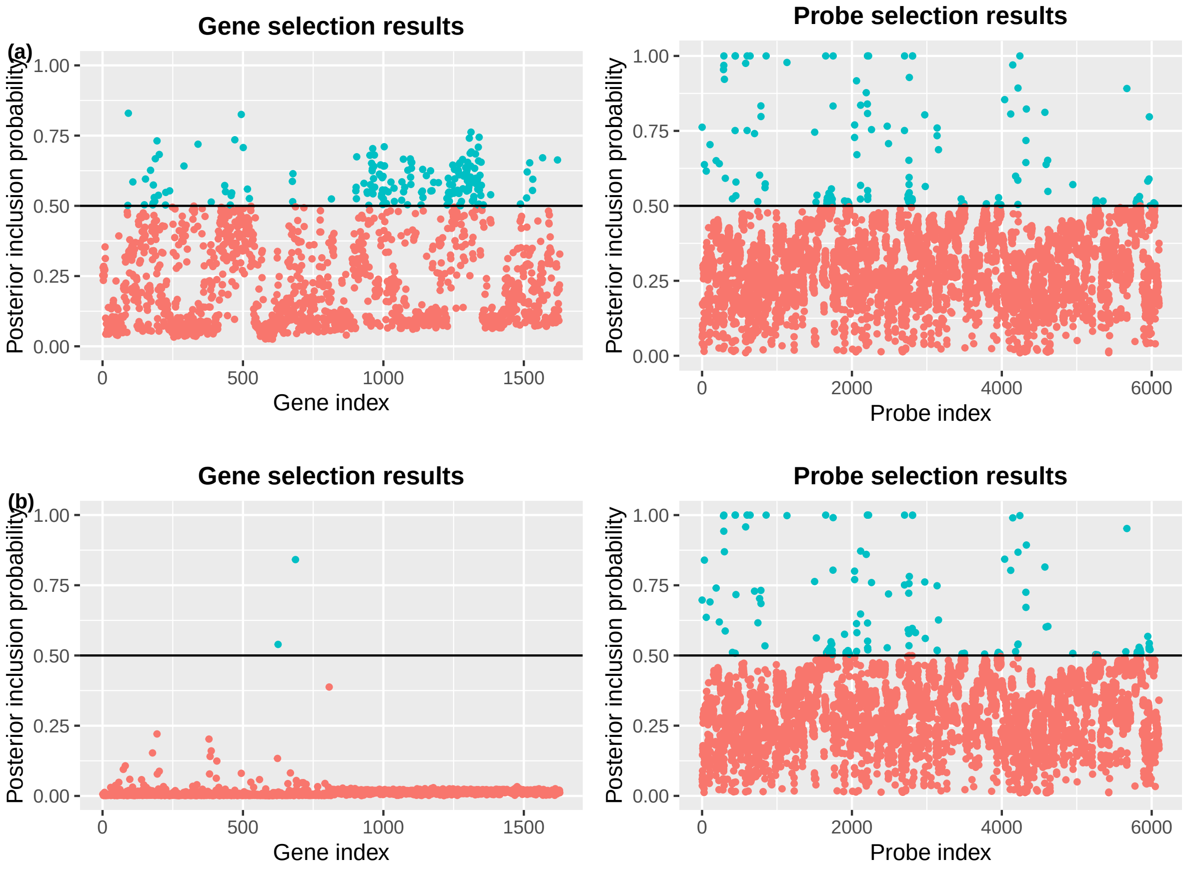

Figure S8: KIRC study: (a) Gene selection results and methylation probe selection results of the proposed method; (b) Gene selection results and methylation probe selection results of the proposed method without MRF prior.

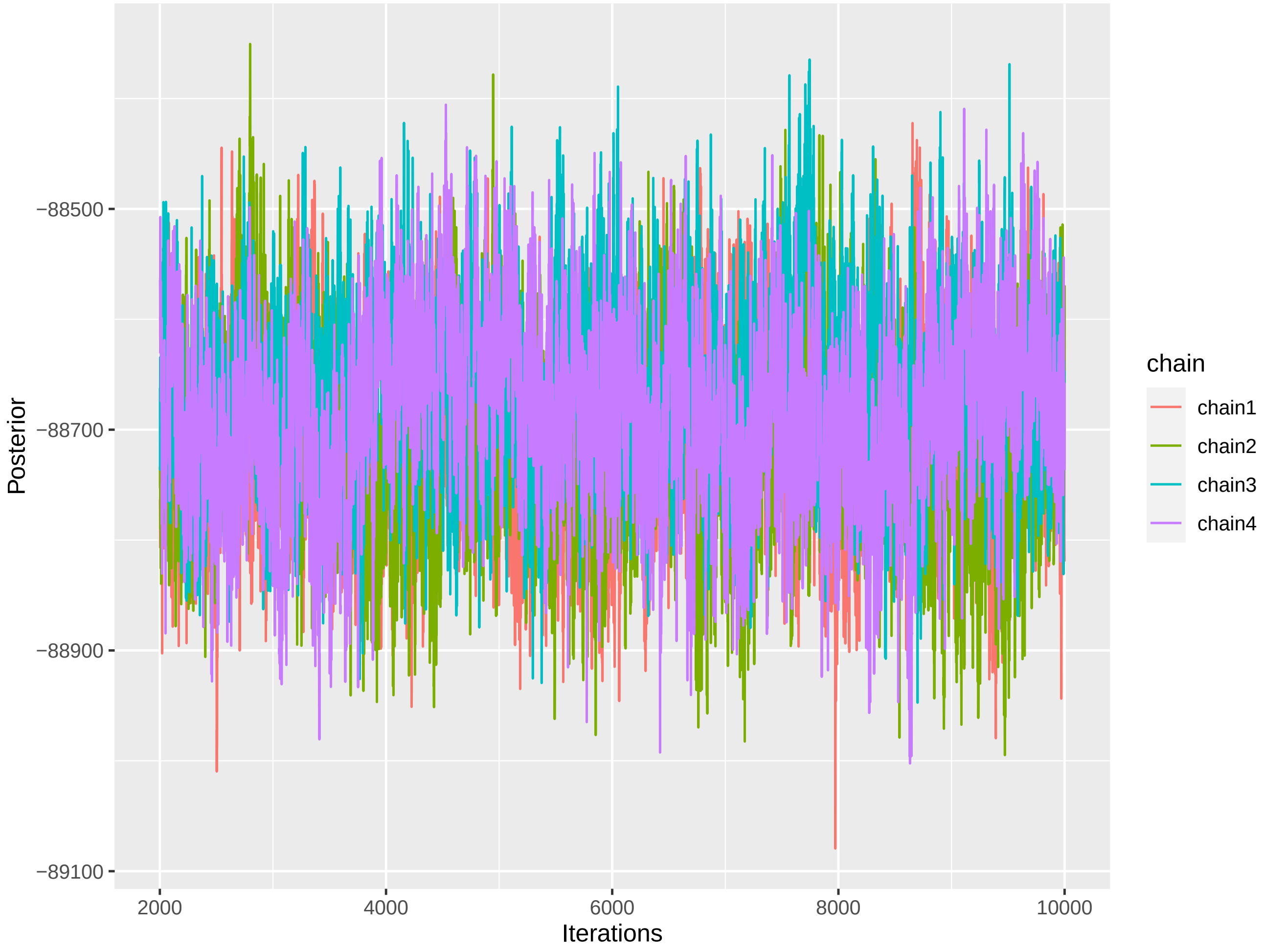

Figure S9: LUAD study: Trace plot of posterior for four different chains.

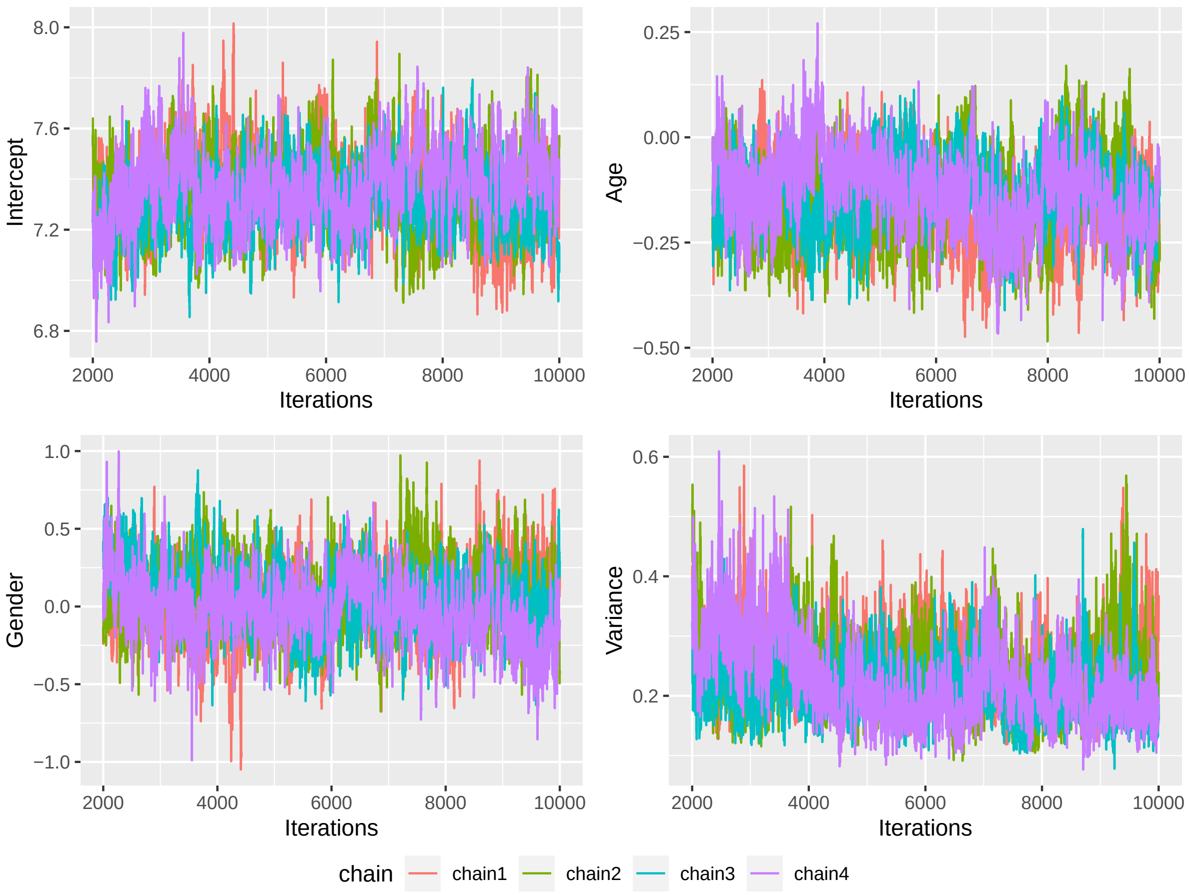

Figure S10: LUAD study: Trace plot of parameters in the four different chains.

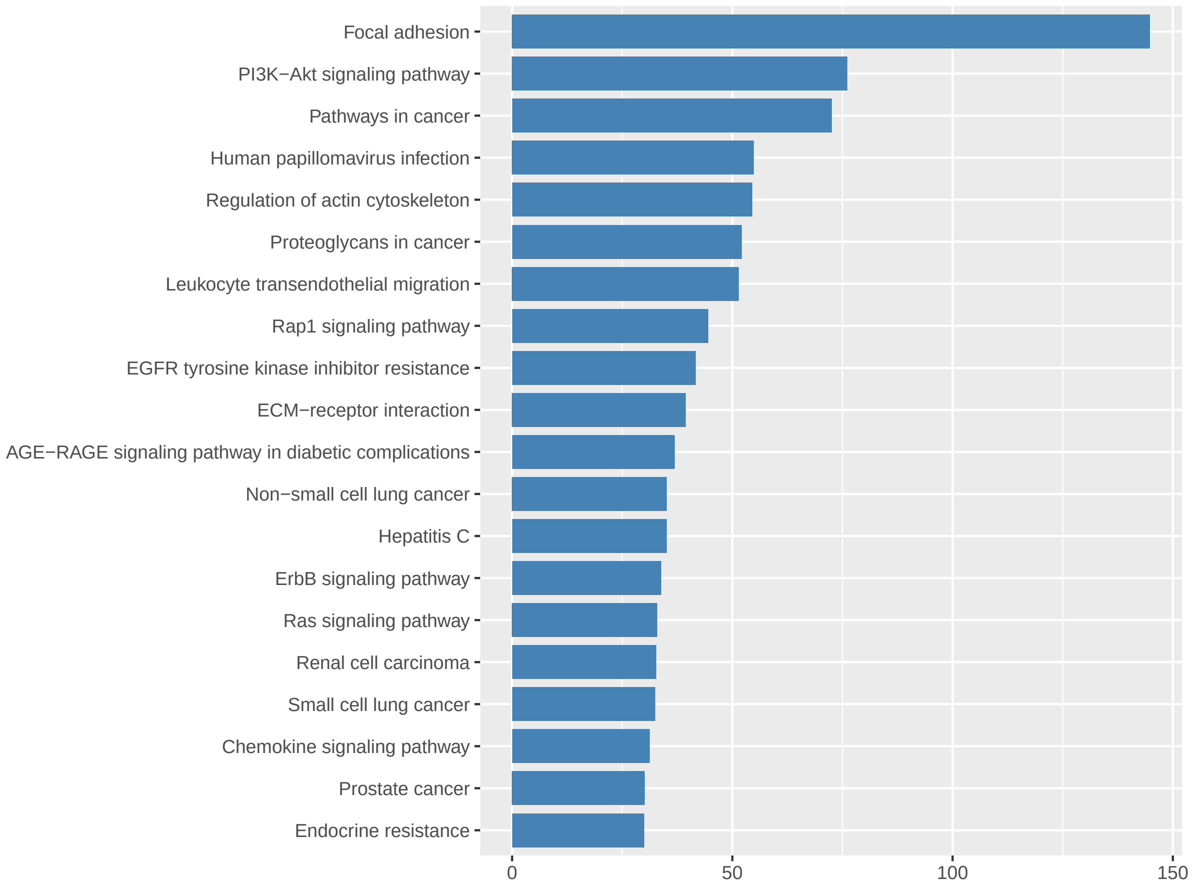

Figure S11: KIRC study: KEGG pathway enrichment analysis results for identified genes by NetMIM

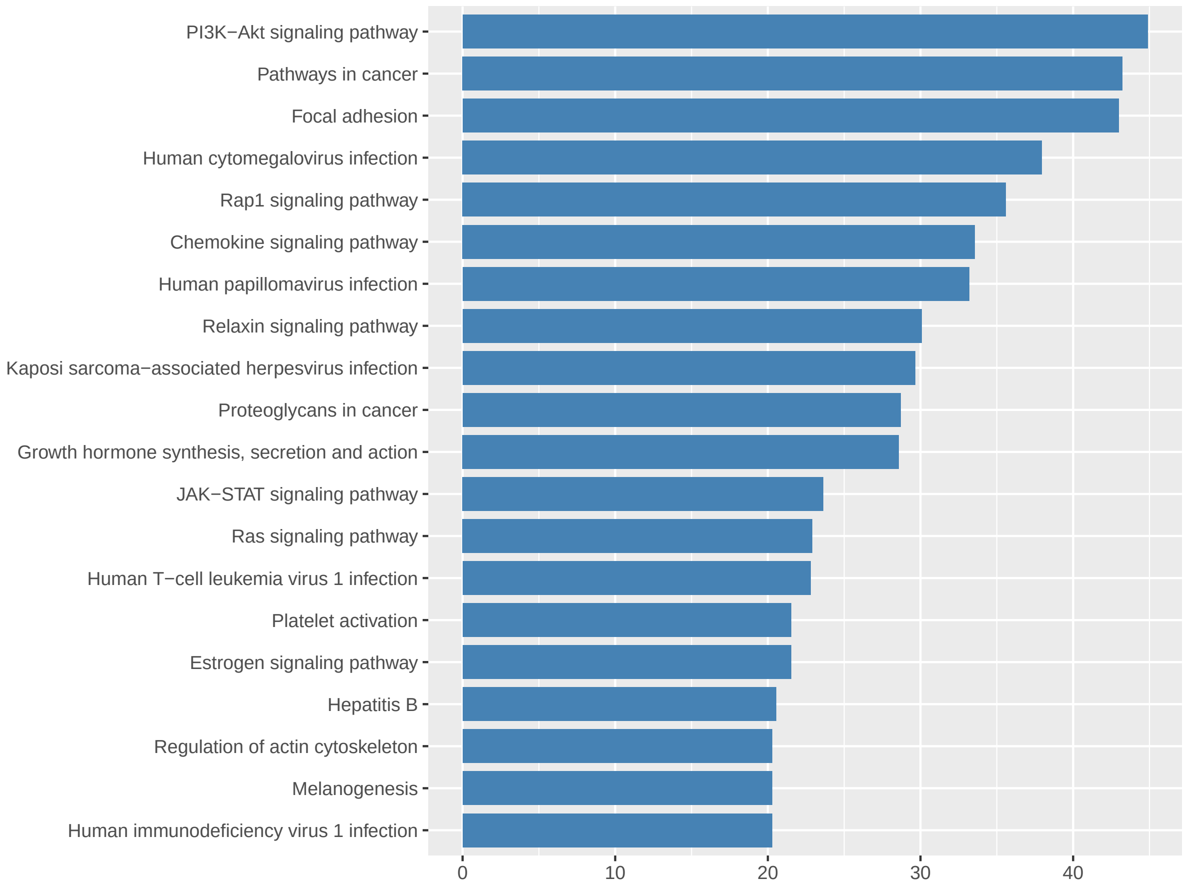

Figure S12: LUAD study: KEGG pathway enrichment analysis results for identified genes by NetMIM
